# Supplementary material for: Single-cell transcriptomic analysis of mouse neocortical development
Source: Nat Commun. 2019 Jan 11;10:134. doi: 10.1038/s41467-018-08079-9 (PMC6329831; doi:10.1038/s41467-018-08079-9)
Supplement: Supplementary file 1 — Supplementary Information [file 41467_2018_8079_MOESM1_ESM.pdf]

Single-cell transcriptomic analysis of mouse neocortical development

Loo and Simon, *et al*.

Supplementary Information

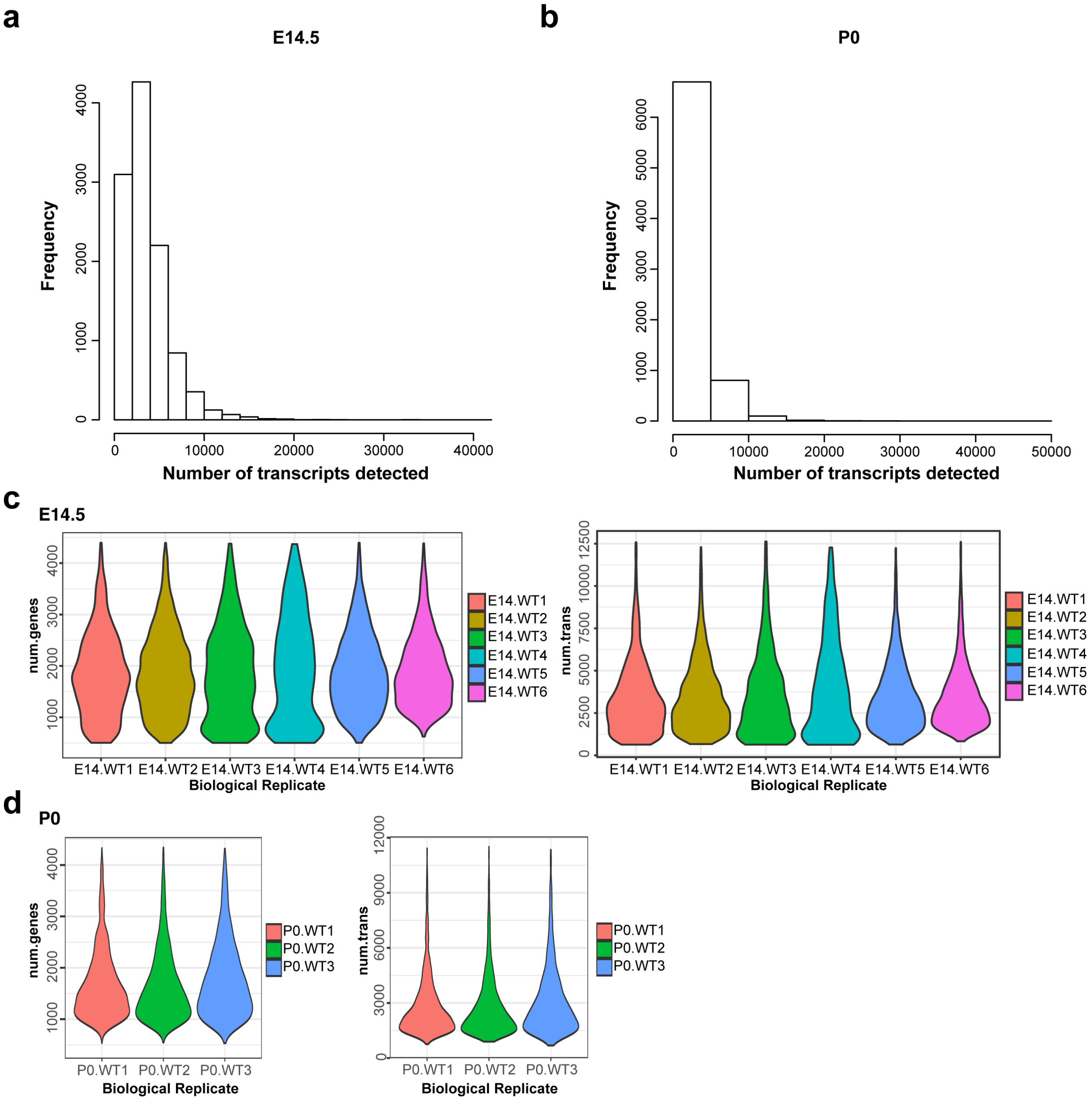

Supplementary Figure 1. **Quality statistics of E14.5 and P0 Drop-seq data**. ***a*–b**. Histogram showing total number of transcripts detected in E14.5 (**a**) and P0 (**b**) samples. **c–d**. Violin plots of total number of genes (left) and total number of transcripts (right) detected for each biological replicate for E14.5 (**c**) and P0 (**d**) samples.

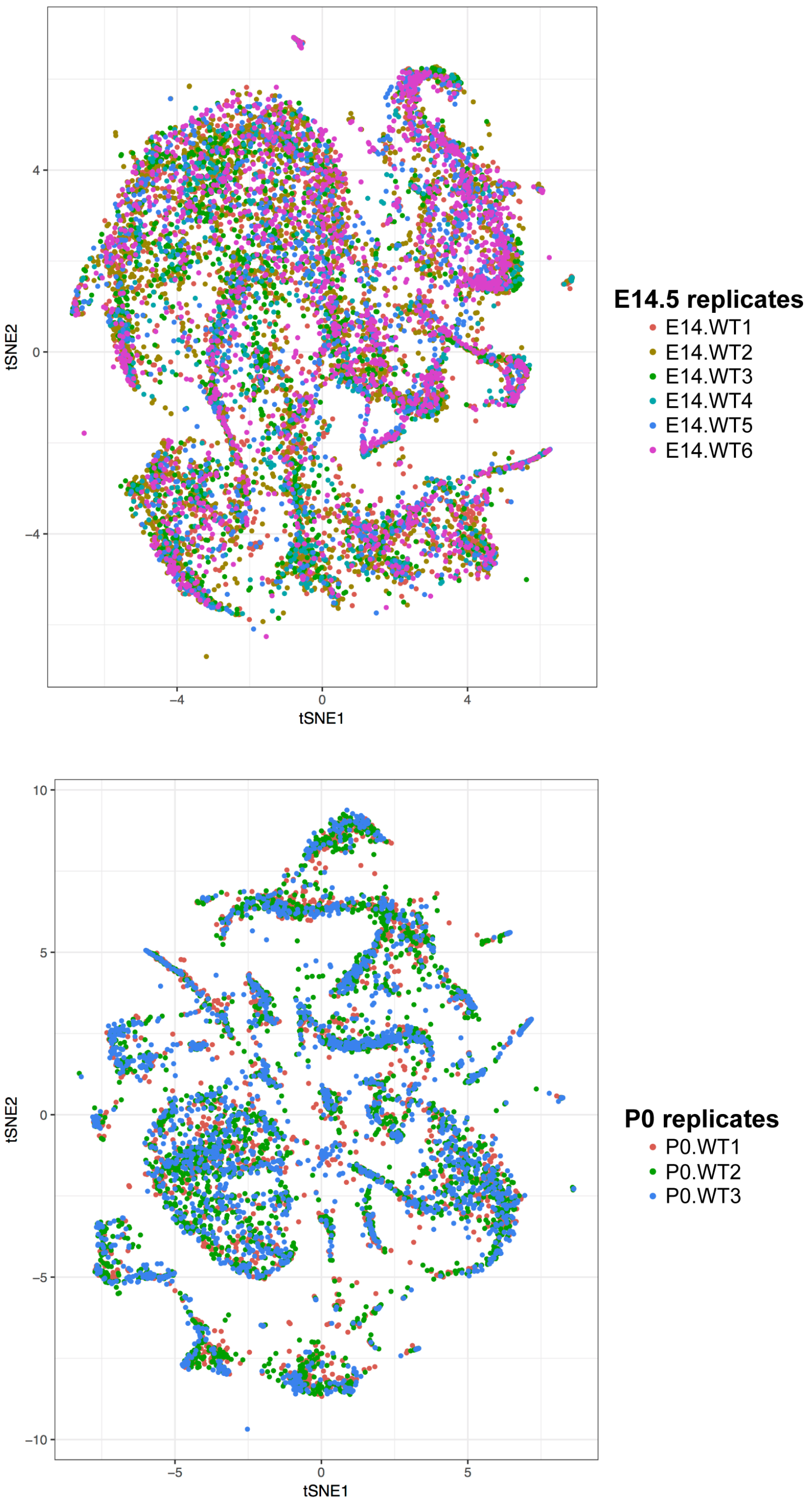

Supplementary Figure 2. Drop-seq expression data from all replicates visualized by tSNE. tSNE visualization of significant principal components, where points are labeled by their biological replicate for E14.5 (top) and P0 (bottom).

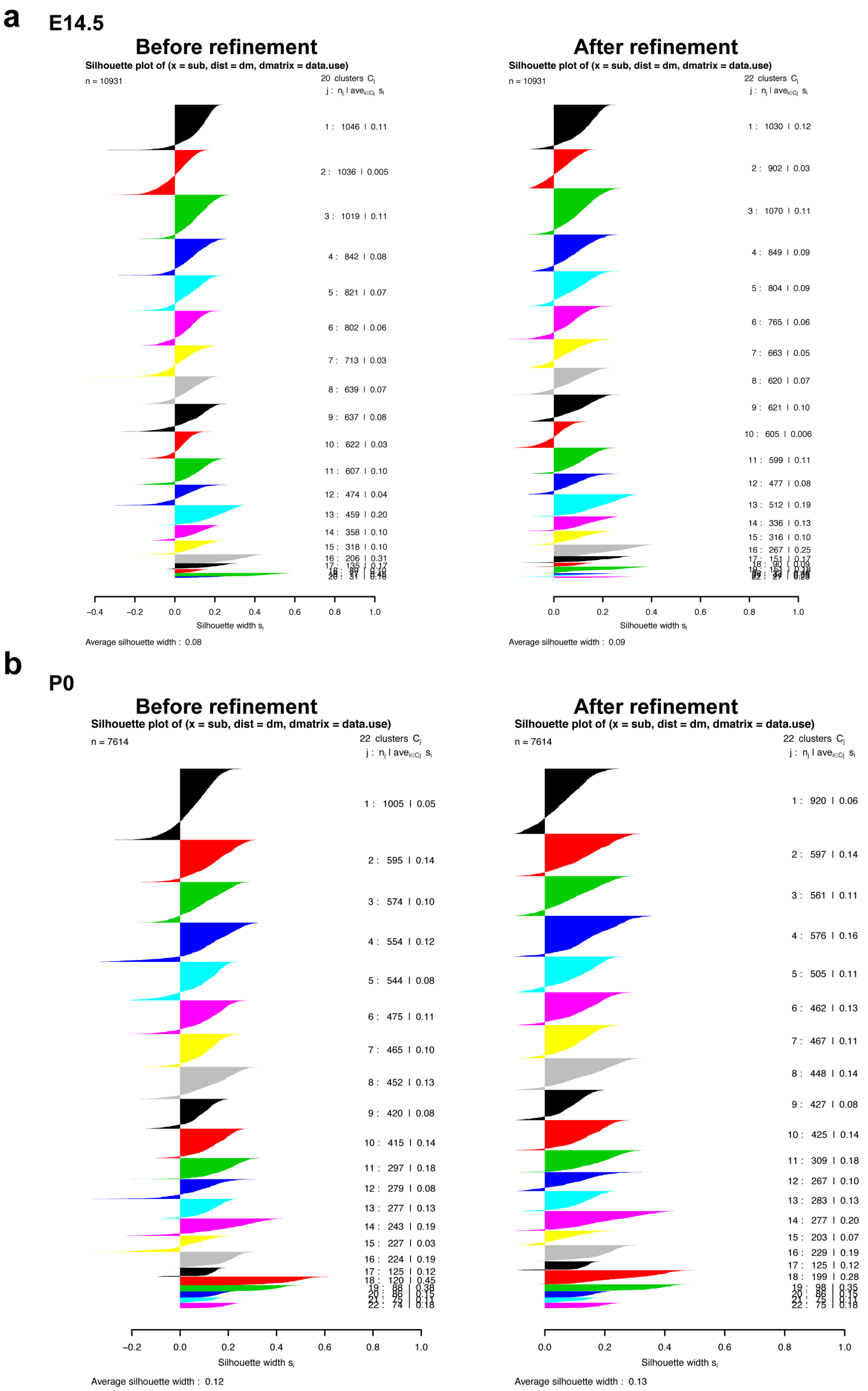

Supplementary Figure 3. Cluster refinement methodology improves cluster assignments and robustness. Silhouette width plots before (left) and after (right) running the cluster refinement method for E14.5 (**a**) and P0 (**b**) samples. Each row represents one cell, and the silhouette widths were determined using Spearman correlation distances. Positive values indicate a robust cluster assignment. The method allows some cluster outliers to form their own novel clusters, which happens twice in the E14.5 samples.

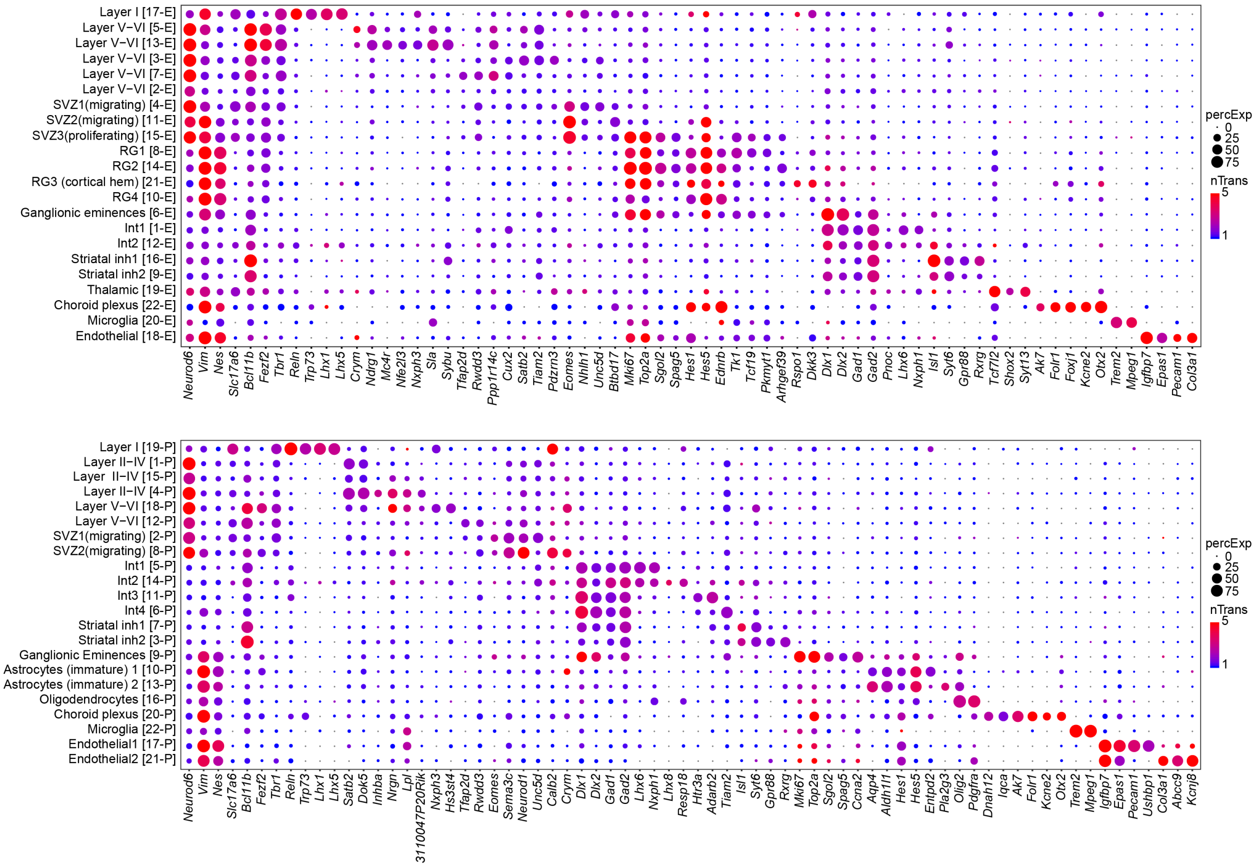

Supplementary Figure 4. Functional characterization of cell types using expression of known and novel marker genes. Dotplot representations of expression levels and proportion of cells with detected transcripts for markers of each cell type for each age, E14.5 (top), and P0 (bottom). Large and red circles indicate expression was high in a large proportion of cells in that cluster, small and blue circles indicate expression was low and detected in few cells in that cluster.

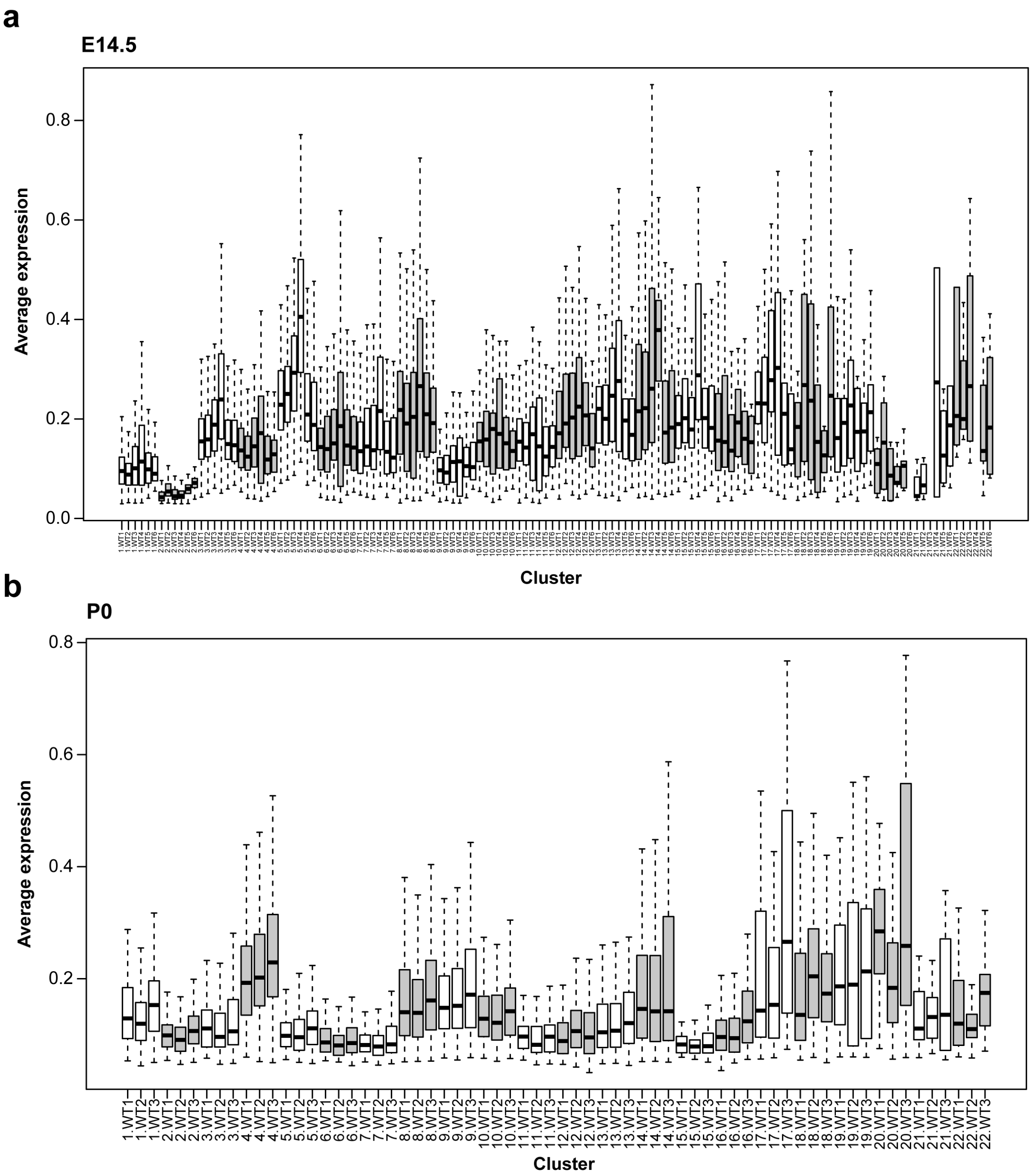

Supplementary Figure 5. Cross-replicate comparison of library quality, separated by cluster. Box-and-whisker plots depicting the distribution of the average expression values for each biological replicate for each cluster, for E14.5 (**a**), and P0 (**b**). Expression values for all genes for a given biological replicate were averaged within each cluster. Variability cluster-to-cluster is expected, as different cell types may have differing numbers of transcripts expressed, however, variability across replicates within a given cluster is limited, suggestive of consistent representation and strong reproducibility.

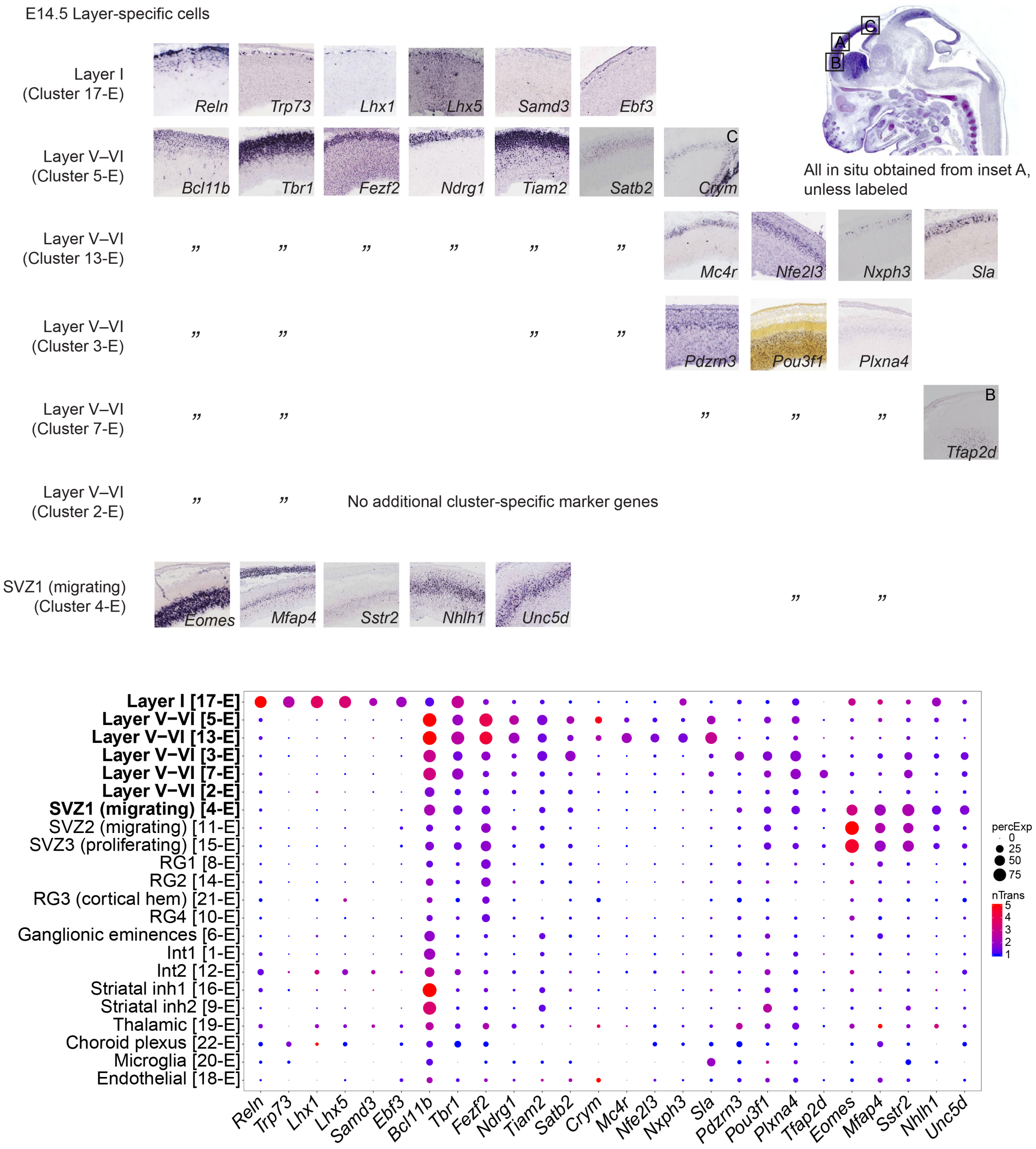

Supplementary Figure 6. Validation of marker gene expression in E14.5 layer-specific cells. RNA *in situ* hybridization images were obtained from E14.5 cerebral cortex, provided by Eurexpress online database (www.eurexpress.org) except *Pou3f1* obtained from Allen Institute for Brain Science (www.developingmouse.brain-map.org). Layer I (17-E) expresses canonical Cajal-Retzius cell markers *Reln*, *Trp73*, *Lhx1* and *Lhx5*. Layer V-VI clusters (5-E, 13-E, 3-E, 7-E and 2-E) express deep layer markers, *Bcl11b* (also known as *Ctip2) and Tbr1.* Expression of *Satb2* in clusters 5-E, 13-E and 3-E suggests eventual differentiation into upper layer callosal projection neurons. SVZ1 (4-E) expresses *Eomes* (also known as *Tbr2*), *Sstr2*, a migrational marker, and *Unc5d*, a netrin receptor involved in cell migration. Dotplot representations of expression levels and proportion of cells with detected transcripts for markers of each cell type. Y axis denotes cluster type; X axis denotes gene name. See **Supplementary Data 2** for annotation of selected markers.

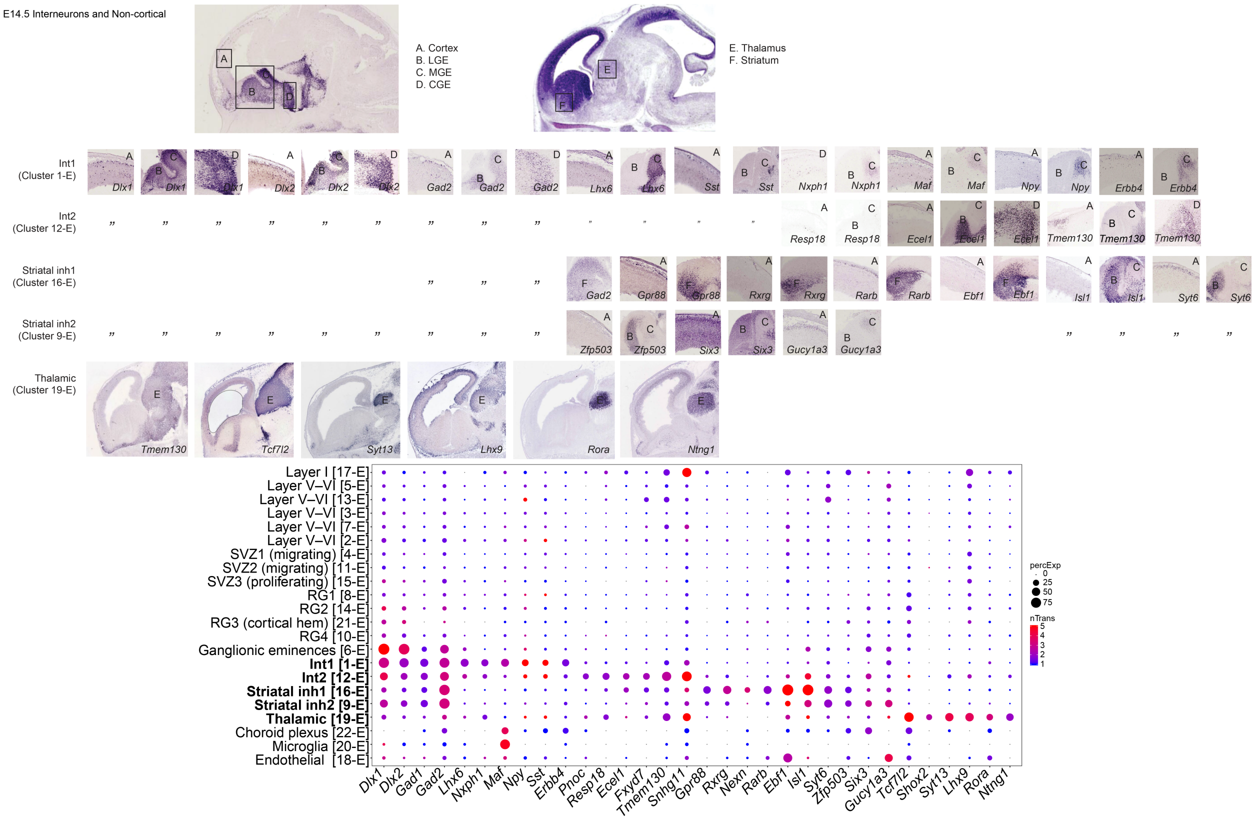

Supplementary Figure 7. Validation of marker gene expression in E14.5 interneurons and non-cortical cells. RNA *in situ* hybridization images were obtained from E14.5 cerebral cortex, ganglionic eminences, striatum and thalamus, provided by Eurexpress online database (www.eurexpress.org). The largest cortical interneuron cluster, Int1 (9.41% of total population, cluster 1-E) expresses *Lhx6*, a transcription factor for MGE-derived cortical Parvalbumin (PV) and Somatostatin (Sst) interneurons. A smaller cluster, Int2 (4.31% of total population, cluster 12-E), expresses lower levels of Lhx6 and RNA i*n situ* expression of *Resp18*, *Ecel1* and *Tmem180* indicate a second distinct population of PV and Sst cortical interneurons. 16-E and 9-E express *Isl1*, a lateral ganglionic eminence (LGE) marker. A subset of these LGE-derived GABAergic neurons are possible cortical interneurons (reported in Anderson *et al*., *Development*, 2001). However, 16-E expresses striatal markers *Gpr88*, *Rxrg* and *Rarb* while 9-E expresses *Zfp503*, a marker of developing striatum and are therefore designated as Striatal inh1 and 2. A small population of cells (1.28% of total population, cluster 19-E) express thalamic markers *Tcf7l2*, *Shox2*, *Syt13*, *Lhx9*, *Rora* and *Ntng1*. Dotplot representations of expression levels and proportion of cells with detected transcripts for markers of each cell type. Y axis denotes cluster type; X axis denotes gene name. See **Supplementary Data 2** for annotation of selected markers.

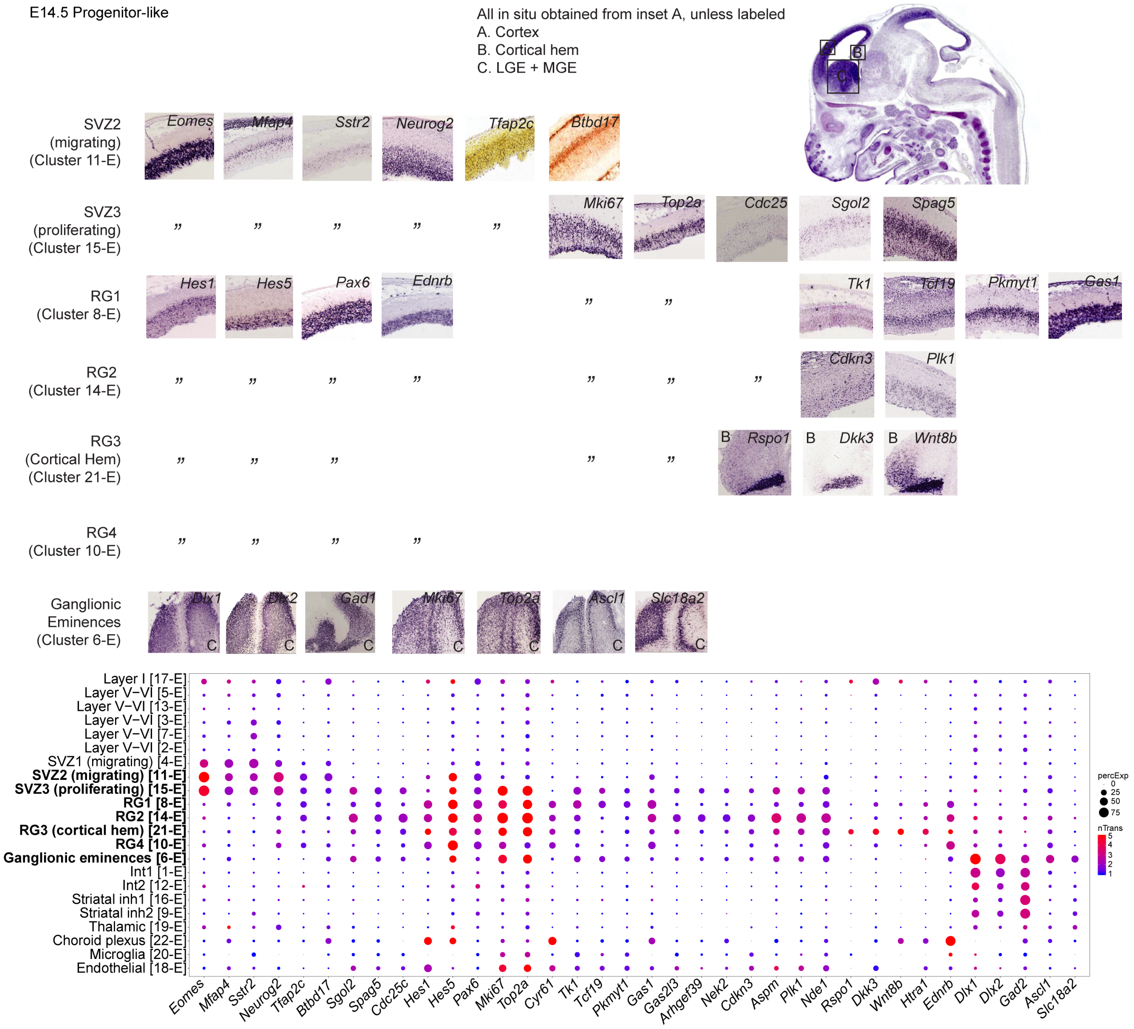

Supplementary Figure 8. Validation of marker gene expression in E14.5 progenitor-like cells. RNA *in situ* hybridization images were obtained from E14.5 cerebral cortex, cortical hem and ganglionic eminences, provided by Eurexpress online database (www.eurexpress.org) except *Tfap2c* obtained from Allen Institute for Brain Science (www.developingmouse.brain-map.org) and *Btbd17* from GENSAT online database (www.gensat.org). SVZ2 (11-E) expresses *Sstr2*, a migrational marker and *Neurog2*, which initiates delamination of radial glia and neuronal lineage commitment. SVZ3 (15-E), RG1 (8-E), RG2 (14-E), RG3 (21-E) and ganglionic eminences (6-E) express *Mki67* and *Top2a*, markers of cell proliferation. RG1–4 express radial glia markers *Hes1*, *Hes5* and *Pax6,* while RG3 specifically expresses *Rspo1*, *Dkk3*, and *Wnt8b*, markers of cortical hem. Ganglionic eminences (6-E) express various GABAergic markers such as *Dlx1*, *Dlx2* and *Gad2*. Dotplot representations of expression levels and proportion of cells with detected transcripts for markers of each cell type. Y axis denotes cluster type; X axis denotes gene name. See **Supplementary Data 2** for annotation of selected markers.

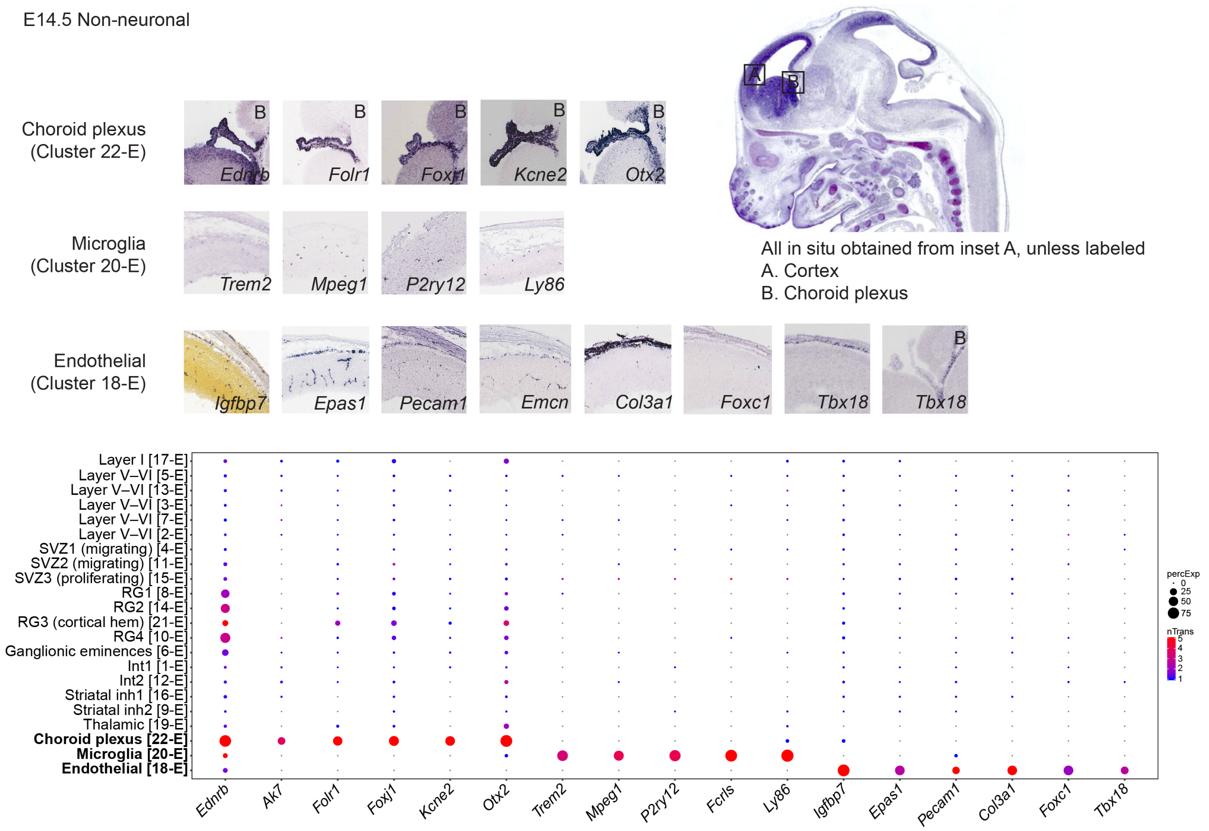

Supplementary Figure 9. Validation of marker gene expression in E14.5 non-neuronal cells. RNA *in situ* hybridization images were obtained from E14.5 cerebral cortex and choroid plexus, provided by Eurexpress online database (www.eurexpress.org) except *Igfbp7* obtained from Allen Institute for Brain Science (www.developingmouse.brain-map.org). Cluster 22-E expresses choroid plexus markers *Folr1*, *Foxj1*, *Kcne2* and *Otx2*. Cluster 20-E expresses microglial markers *Trem2*, *Mpeg1*, *P2ry12* and *Ly86*. Cluster 18-E expresses endothelial markers *Igfbp7*, *Epas1* and *Pecam1*. Dotplot representations of expression levels and proportion of cells with detected transcripts for markers of each cell type. Y axis denotes cluster type; X axis denotes gene name. See **Supplementary Data 2** for annotation of selected markers.

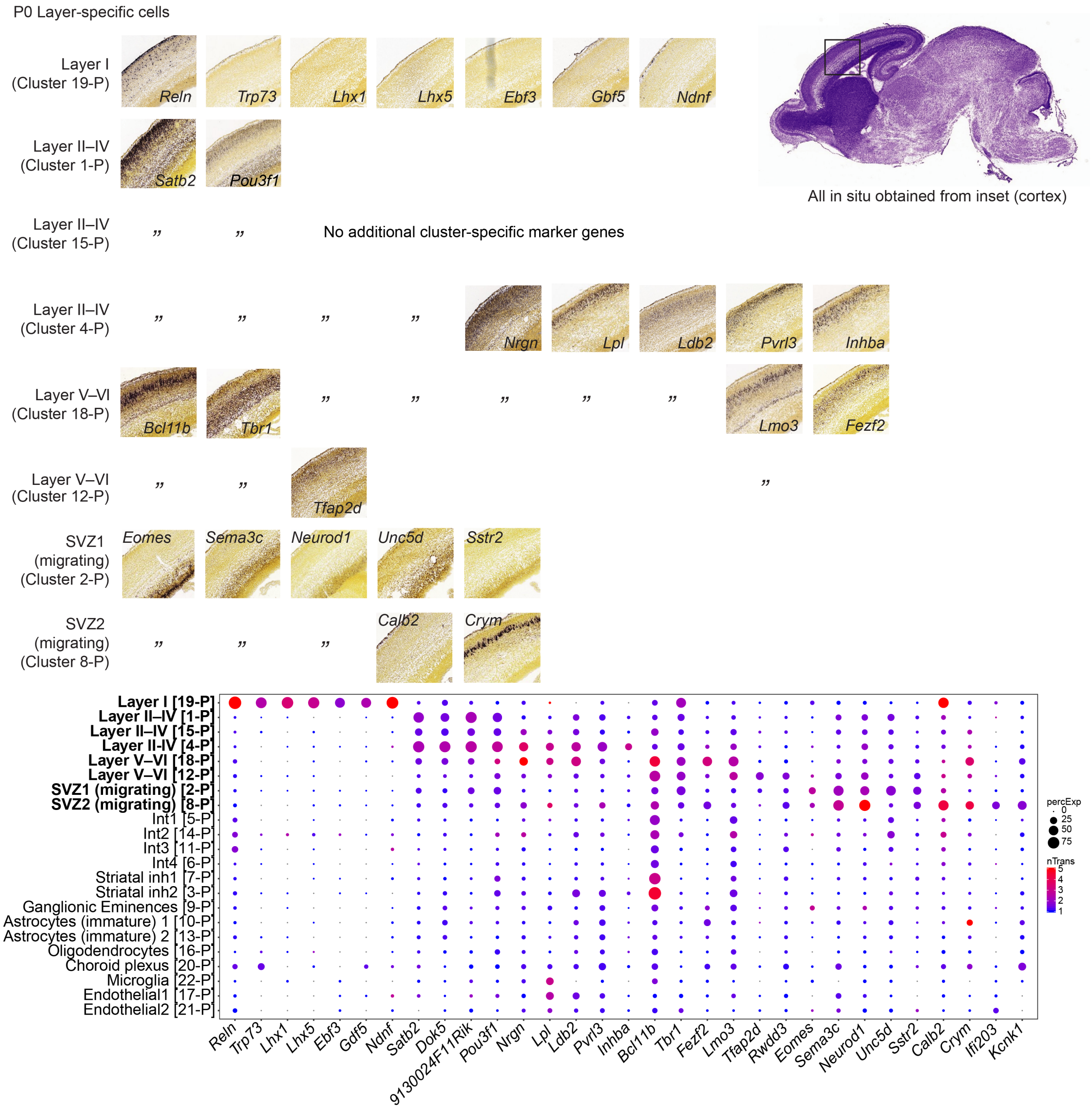

Supplementary Figure 10. Validation of marker gene expression in P0 layer-specific cells. RNA *in situ* hybridization images were obtained from E18.5 cerebral cortex, provided by Allen Institute for Brain Science (www.developingmouse.brain-map.org). Layer I (cluster 19-P) expresses Cajal-Retzius markers *Reln*, *Trp73*, *Lhx1* and *Lhx5*. Layer II–IV (clusters 1-P, 15-P, 4-P) expresses *Satb2*, a marker for upper layer callosal projection neurons and *Pou3f1*, a migrational marker observed in cluster 3-E (**Supplementary Fig. 6**). Deeper layer clusters 18-P and 12-P express *Bcl11b* and *Tbr1*. 18-P is designated layer V–VI as it expresses *Fezf2*, a layer V-specific corticospinal neuron marker. SVZ1 (2-P) and SVZ2 (8-P) express *Eomes* (*Tbr2*) and *Sema3c*, a marker of migrating newborn neurons. Dotplot representations of expression levels and proportion of cells with detected transcripts for markers of each cell type. Y axis denotes cluster type; X axis denotes gene name. See **Supplementary Data 2** for annotation of selected markers.

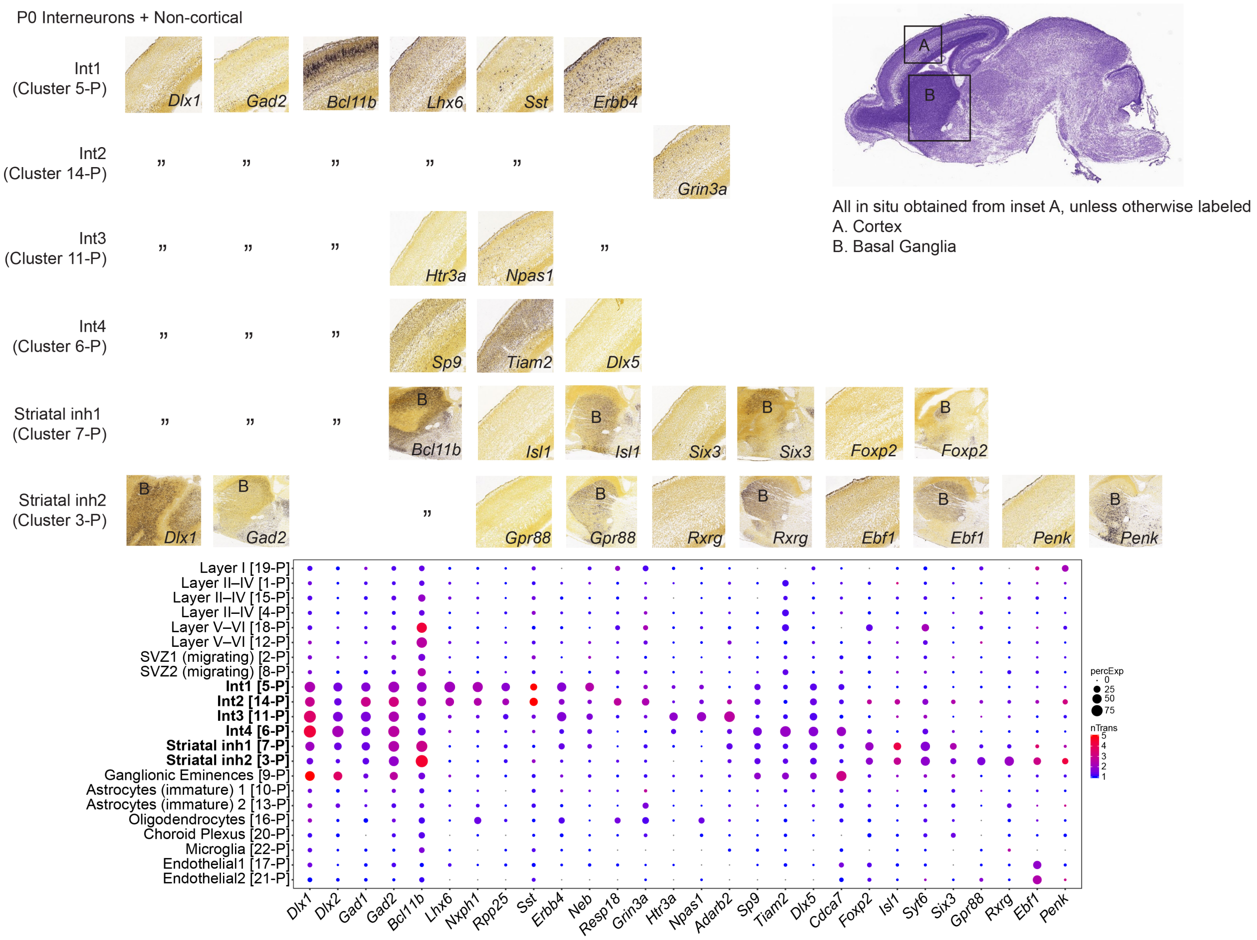

Supplementary Figure 11. Validation of marker gene expression in P0 interneurons and non-cortical cells. RNA *in situ* hybridization images were obtained from E18.5 cerebral cortex, basal ganglia and preoptic area, provided by Allen Institute for Brain Science (www.developingmouse.brain-map.org). Int1 (5-P), Int2 (14-P), Int3 (11-P), Int4 (6-P), Striatal inh1 (7-P) and Striatal inh2 (3-P) express GABAergic markers *Dlx1*, *Dlx2*, *Gad1* and *Gad2*. Int1 and Int2 express *Lhx6* and *Nxph1,* markers of PV and Sst^{+} interneuron precursors, while Int4 expresses *Cdca7*, a marker expressed in a subset of PV and Sst^{+} interneurons. Int3 expresses *Htr3a*, *Npas1* and *Adarb2*, markers of VIP cortical interneurons. Striatal inh1 and 2 express striatal markers *Isl1* and *Gpr88* respectively. Dotplot representations of expression levels and proportion of cells with detected transcripts for markers of each cell type. Y axis denotes cluster type; X axis denotes gene name. See **Supplementary Data 2** for annotation of selected markers.

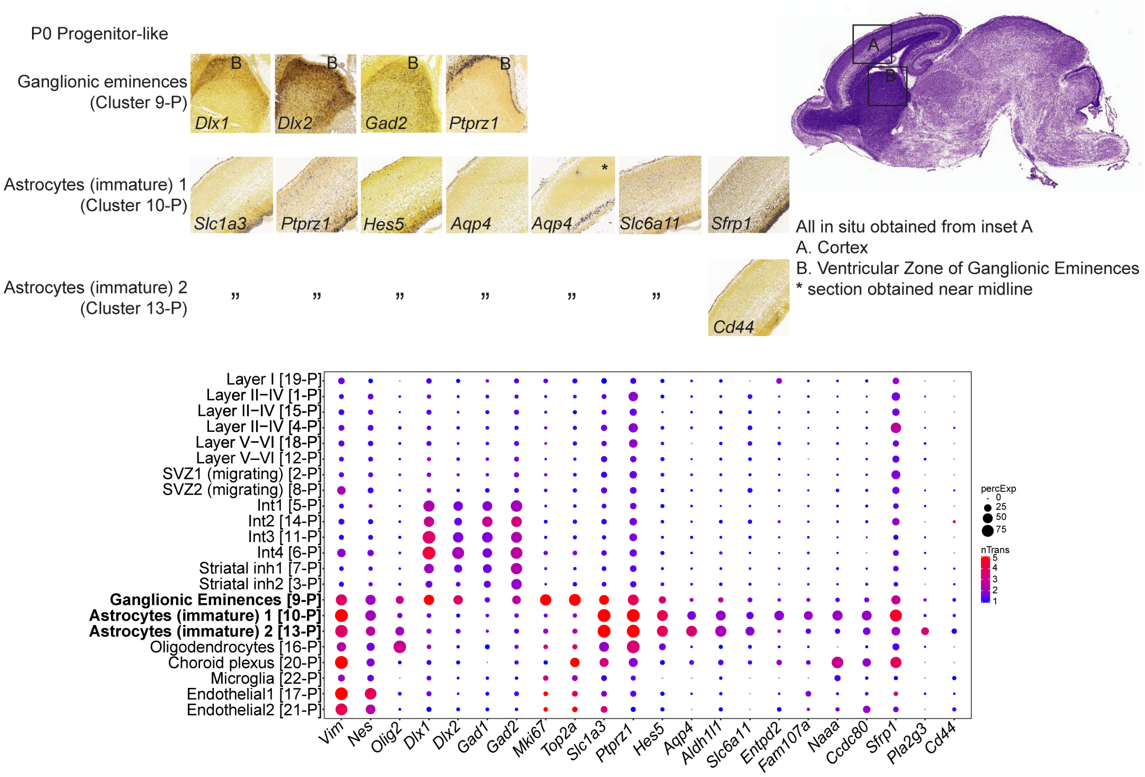

Supplementary Figure 12. Validation of marker gene in P0 progenitor-like cells. RNA *in situ* hybridization images were obtained from E18.5 cerebral cortex and ganglionic eminence, provided by Allen Institute for Brain Science (www.developingmouse.brain-map.org). Ganglionic eminences (Cluster 9-P) express GABAergic markers *Dlx1*, *Dlx2* and *Gad2* and proliferation markers *Mki67* and *Top2a*. Astrocytes (immature) 1 (10-P) and 2 (13-P) express astrocytic markers *Ptprz1*, *Aqp4* and *Aldh1l1* while maintaining some expression of radial glia marker *Hes5*. Dotplot representations of expression levels and proportion of cells with detected transcripts for markers of each cell type. Y axis denotes cluster type; X axis denotes gene name. See **Supplementary Data 2** for annotation of selected markers.

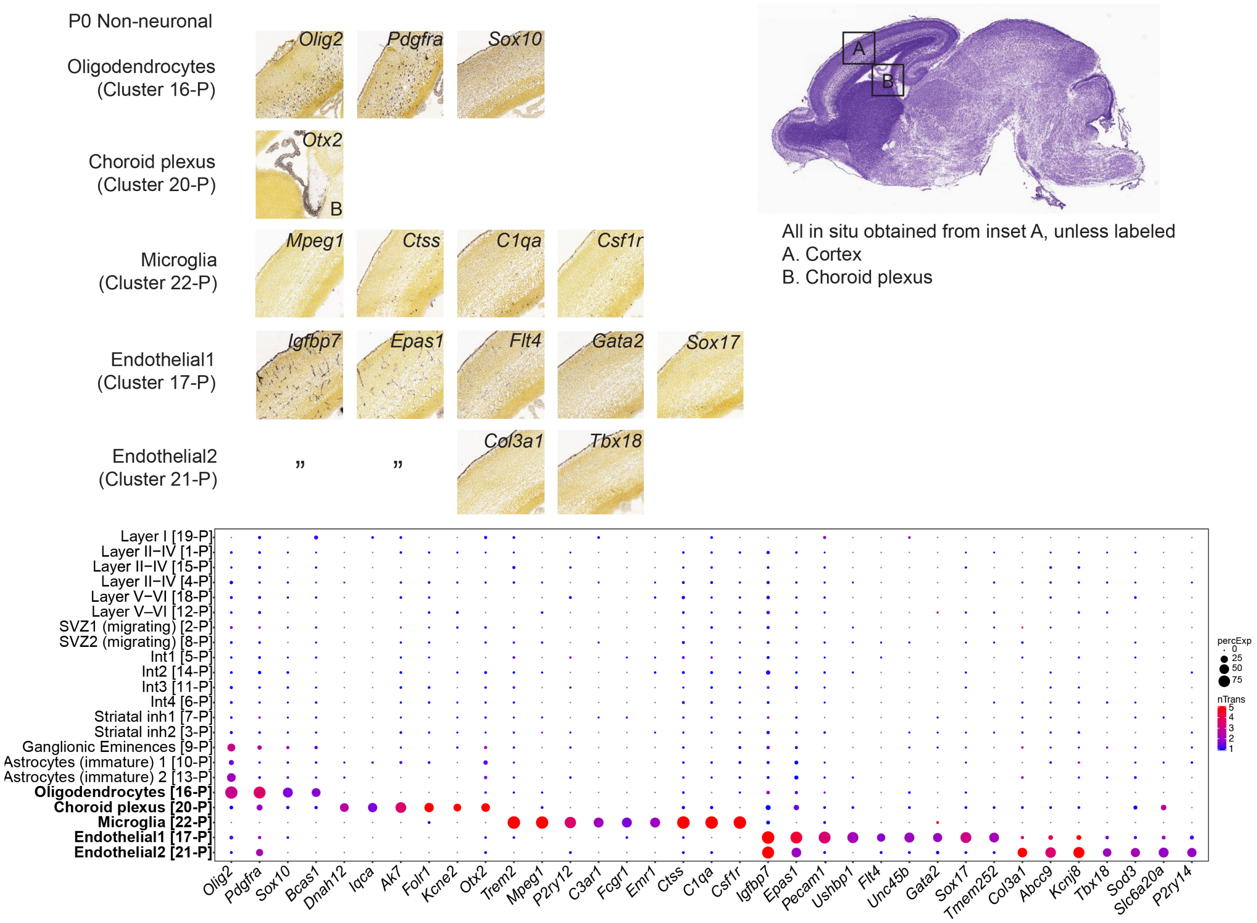

Supplementary Figure 13. Validation of marker gene expression in P0 non-neuronal cells. RNA *in situ* hybridization images were obtained from E18.5 cerebral cortex and choroid plexus, provided by Allen Institute for Brain Science (www.developingmouse.brain-map.org). Cluster 16-P expresses oligodendrocyte markers *Olig2* and *Pdgfra*. Cluster 20-P expresses choroid plexus markers *Ak7*, *Folr1*, *Kcne2* and *Otx2*. Cluster 22-P expresses microglial markers *Mpeg1*, *Ctss*, *C1qa* and *Csf1r*. Clusters 17-P and 21-P express endothelial markers *Igfbp7* and *Epas1*. Dotplot representations of expression levels and proportion of cells with detected transcripts for markers of each cell type. Y axis denotes cluster type; X axis denotes gene name. See **Supplementary Data 2** for annotation of selected markers.

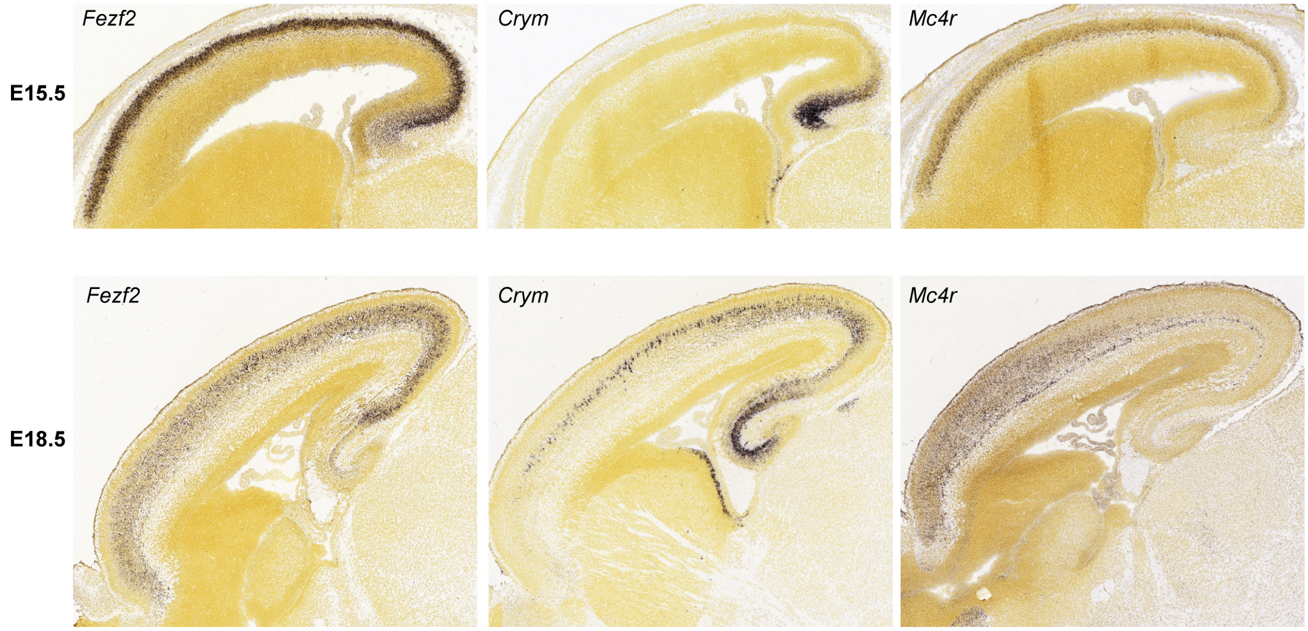

Supplementary Figure 14. Validation of area-specific expression of *Crym* and *Mc4r* in E15.5 and E18.5 cortex. RNA *in situ* hybridization images were obtained from E15.5 and E18.5 cerebral cortex, provided by Allen Institute for Brain Science (www.developingmouse.brain-map.org). Upper-layer neurons marked by *Fezf2* express *Crym* (caudally) or *Mc4r* (rostrally).

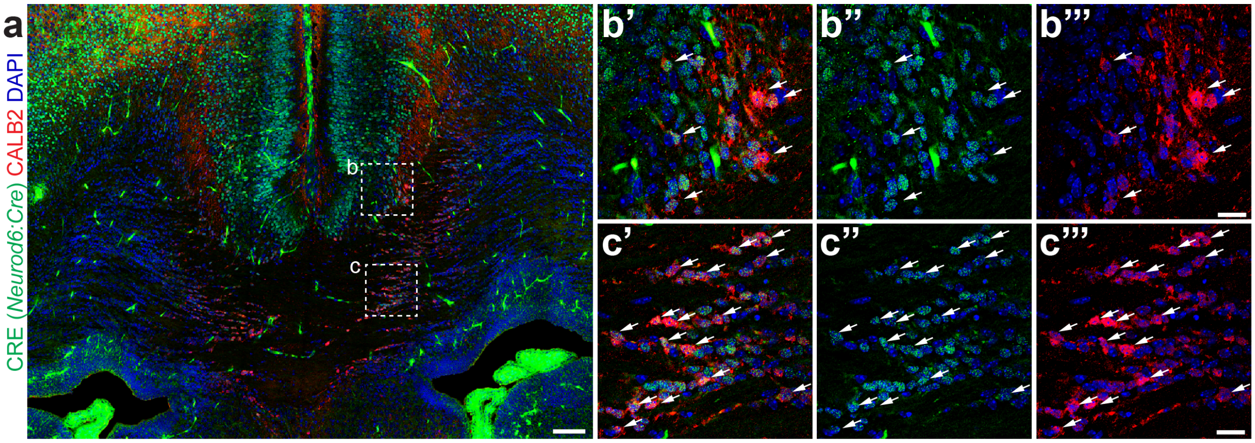

Supplementary Figure 15. Empirical validation of NEUROD6 and CALB2 co-labeling in the P0 cortex. Coronal sections of the P0 cortex from *Neurod6-CRE* mice were stained with antibodies against CRE (green), CALB2 (calretinin, red), and DAPI (blue). Cells expressing both CRE and CALB2 were observed in the cortical plate (**a**–**b**) and corpus callosum (**a, c**). In the corpus callosum, $CRE^{+}CALB2^{+}$ cells were distributed along chain-like cell stripes (**a, c**). Higher resolution images of insets **b** and **c** were shown in (**b'**, **b''** and **b'''**) and (**c'**, **c''** and **c'''**), respectively. Examples of $CRE^{+}CALB2^{+}$ cells are indicated by arrowheads. Scale bar in panel **a**, 200 μm; scale bars in **b'''** and **c'''**, 20 μm.

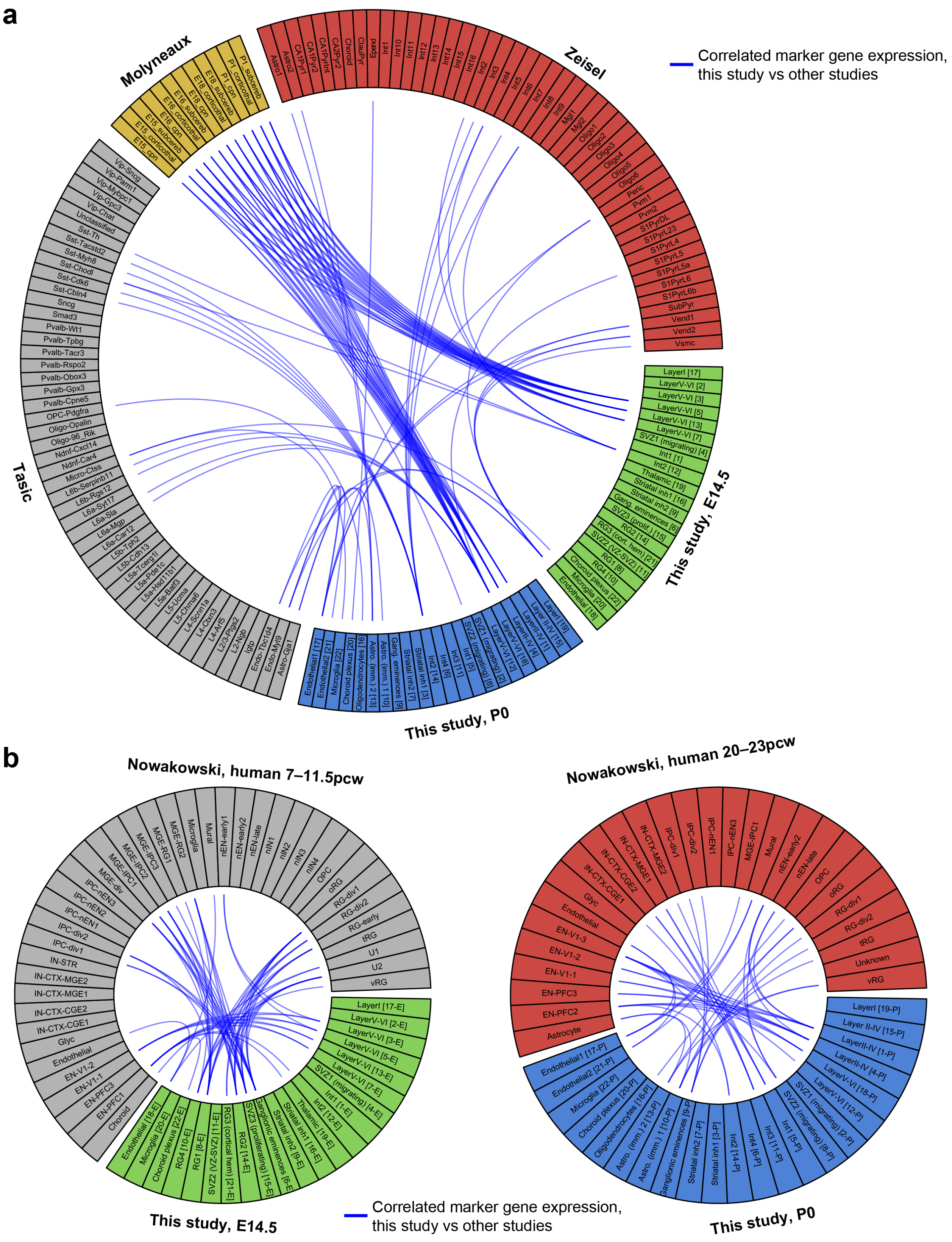
a
Molyneaux
Zeisel
Tasic
This study, E14.5
This study, P0
Correlated marker gene expression, this study vs other studies
P1 subcereb
P1 corticothal
P1 cpn
E18 subcereb
E18 corticothal
E18 cpn
E16 subcereb
E16 corticothal
E16 cpn
E15 subcereb
E15 corticothal
E15 cpn
Astro1
Astro2
CA1Pyr1
CA1Pyr2
CA1PyrInt
CA2Pyr2
Choroid
ClauPyr
Epend
Int1
Int10
Int11
Int12
Int13
Int14
Int15
Int16
Int2
Int3
Int4
Int5
Int6
Int7
Int8
Int9
Mgl1
Mgl2
Oligo1
Oligo2
Oligo3
Oligo4
Oligo5
Oligo6
Peric
Pvm1
Pvm2
S1PyrDL
S1PyrL23
S1PyrL4
S1PyrL5
S1PyrL5a
S1PyrL6
S1PyrL6b
SubPyr
Vend1
Vend2
Vsmc
LayerI [17]
LayerV-VI [2]
LayerV-VI [3]
LayerV-VI [5]
LayerV-VI [13]
LayerV-VI [7]
SVZ1 (migrating) [4]
Int1 [1]
Int2 [12]
Thalamic [19]
Striatal inh1 [16]
Striatal inh2 [9]
Gang. eminences [6]
SVZ3 (prolif.) [15]
RG2 [14]
RG3 (cort. hem) [21]
SVZ2 (VZ-SVZ) [11]
RG1 [8]
RG4 [10]
Choroid plexus [22]
Microglia [20]
Endothelial [18]
LayerI [19]
LayerII-IV [15]
LayerII-IV [1]
LayerII-IV [4]
LayerV-VI [18]
LayerV-VI [12]
SVZ1 (migrating) [2]
SVZ2 (migrating) [8]
Int1 [5]
Int3 [11]
Int4 [6]
Int2 [14]
Striatal inh1 [3]
Striatal inh2 [7]
Gang. eminences [9]
Astro. (imm.) 1 [10]
Astro. (imm.) 2 [13]
Oligodendrocytes [16]
Choroid plexus [20]
Microglia [22]
Endothelial2 [21]
Endothelial1 [17]
Vip-Sncg
Vip-Parm1
Vip-Mybpc1
Vip-Gpc3
Vip-Chat
Unclassified
Sst-Th
Sst-Tacstd2
Sst-Myh8
Sst-Chodl
Sst-Cdk6
Sst-Cbln4
Sncg
Smad3
Pvalb-Wt1
Pvalb-Tpbg
Pvalb-Tacr3
Pvalb-Rspo2
Pvalb-Obox3
Pvalb-Gpx3
Pvalb-Cpne5
OPC-Pdgfra
Oligo-Opalin
Oligo-96_Rik
Ndnf-Cxcl14
Ndnf-Car4
Micro-Ctss
L6b-Serpinb11
L6b-Rgs12
L6a-Syt17
L6a-Sla
L6a-Mgp
L6a-Car12
L5b-Tph2
L5b-Cdh13
L5a-Tcerg1l
L5a-Pde1c
L5a-Hsd11b1
L5a-Batf3
L5-Ucma
L4-Chrna6
L4-Scnn1a
L4-Ctxn3
L4-Arf5
L2/3-Ptgs2
L2-Ngb
Igtp
Endo-Tbc1d4
Endo-Myl9
Astro-Gja1
b
Nowakowski, human 7–11.5pcw
Nowakowski, human 20–23pcw
This study, E14.5
This study, P0
Correlated marker gene expression, this study vs other studies
MGE-RG2
MGE-RG1
MGE-IPC3
MGE-IPC2
MGE-IPC1
MGE-div
IPC-nEN3
IPC-nEN2
IPC-nEN1
IPC-div2
IPC-div1
IN-STR
IN-CTX-MGE2
IN-CTX-MGE1
IN-CTX-CGE2
IN-CTX-CGE1
Glyc
Endothelial
EN-V1-2
EN-V1-1
EN-PFC3
EN-PFC1
Choroid
Microglia
Mural
nEN-early1
nEN-early2
nEN-late
nIN1
nIN2
nIN3
nIN4
OPC
oRG
RG-div1
RG-div2
RG-early
tRG
U1
U2
vRG
LayerI [17-E]
LayerV-VI [2-E]
LayerV-VI [3-E]
LayerV-VI [5-E]
LayerV-VI [13-E]
LayerV-VI [7-E]
SVZ1 (migrating) [4-E]
Int1 [1-E]
Int2 [12-E]
Thalamic [19-E]
Striatal inh1 [16-E]
Striatal inh2 [9-E]
Ganglionic eminences [6-E]
SVZ3 (proliferating) [15-E]
RG2 [14-E]
RG3 (cortical hem) [21-E]
SVZ2 (VZ-SVZ) [11-E]
RG1 [8-E]
RG4 [10-E]
Choroid plexus [22-E]
Microglia [20-E]
Endothelial [18-E]
IN-CTX-MGE2
IN-CTX-MGE1
IN-CTX-CGE2
IN-CTX-CGE1
Glyc
Endothelial
EN-V1-3
EN-V1-2
EN-V1-1
EN-PFC3
EN-PFC2
Astrocyte
IPC-div1
IPC-div2
IPC-nEN1
IPC-nEN3
MGE-IPC1
Mural
nEN-early2
nEN-late
OPC
oRG
RG-div1
RG-div2
tRG
Unknown
vRG
LayerI [19-P]
Layer II-IV [15-P]
LayerII-IV [1-P]
LayerII-IV [4-P]
LayerV-VI [18-P]
LayerV-VI [12-P]
SVZ1 (migrating) [2-P]
SVZ2 (migrating) [8-P]
Int1 [5-P]
Int3 [11-P]
Int4 [6-P]
Int2 [14-P]
Striatal inh1 [3-P]
Striatal inh2 [7-P]
Ganglionic eminences [9-P]
Astro. (imm.) 1 [10-P]
Astro. (imm.) 2 [13-P]
Oligodendrocytes [16-P]
Choroid plexus [20-P]
Microglia [22-P]
Endothelial2 [21-P]
Endothelial1 [17-P]

Supplementary Figure 16. Correlative analyses to other published datasets. a. Correlation of expression profiles between cells identified in this study and those identified by Tasic *et al*., Molyneaux *et al*., and Zeisel *et al*. Gene expression matrices were collapsed by taking mean of all cells within a cluster, then correlations were computed pairwise between all cell types of all studies using the intersection of cell-specific and subcluster-specific genes. Lines were drawn only for comparisons between data from this study and other studies, and when those correlations exceeded a minimum threshold ($r > 0.3$). **b.** Similar analysis as in (**a**) except with cell types and marker genes (orthologs) from Nowakowski *et al.* study of human fetal cells. Threshold for depicting correlation was $r > 0.2$.

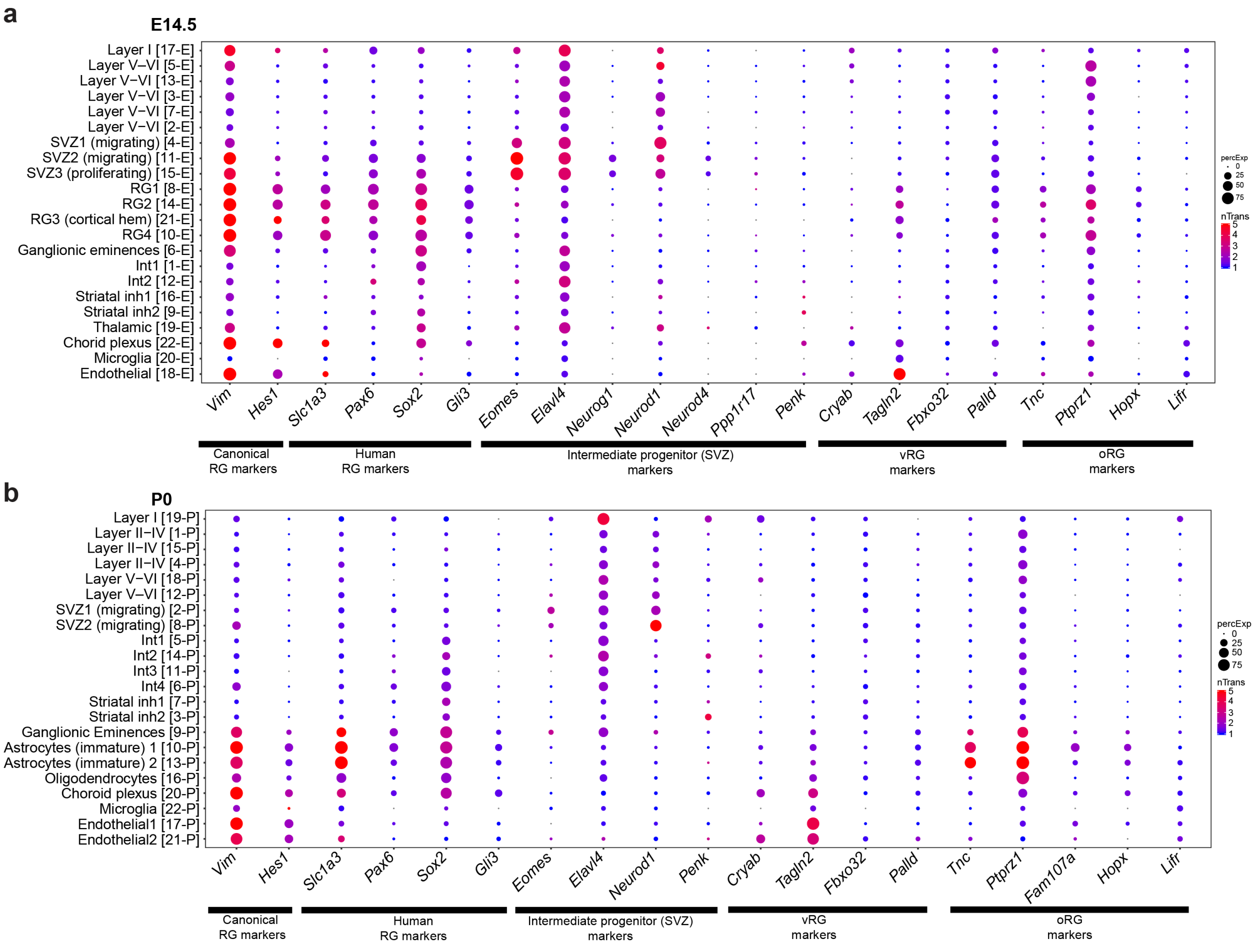

Supplementary Figure 17. Expression of human radial glia markers. Dotplot representations of expression levels and proportion of cells with detected transcripts for markers of human radial glia cell types in E14.5 (**a**) and P0 (**b**) cortex. Y axis denotes cluster type; X axis denotes gene name and radial glia cell type.

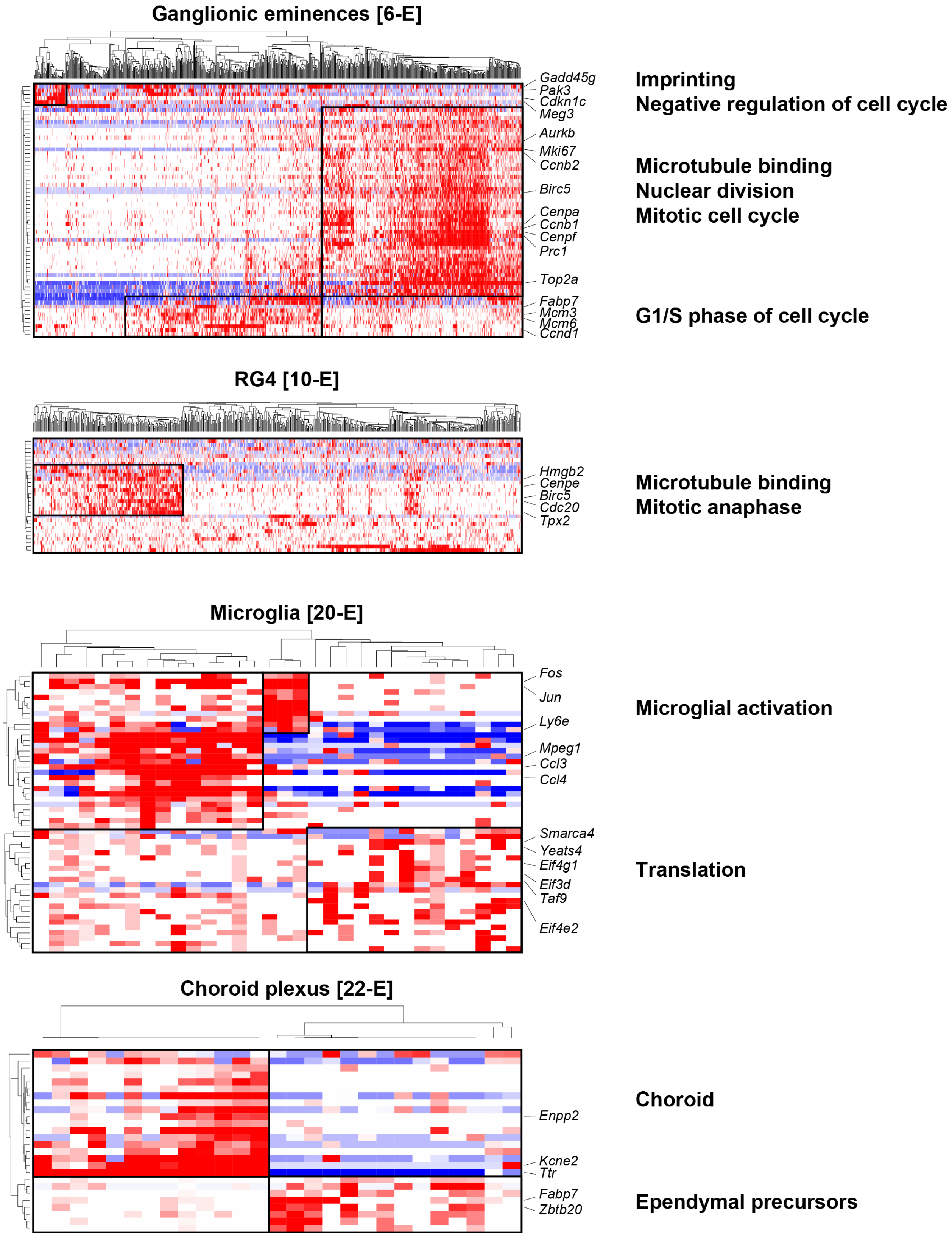

Ganglionic eminences [6-E]
Gadd45g
Pak3
Cdkn1c
Meg3
Aurkb
Mki67
Ccnb2
Birc5
Cenpa
Ccnb1
Cenpf
Prc1
Top2a
Fabp7
Mcm3
Mcm6
Ccnd1
Imprinting
Negative regulation of cell cycle
Microtubule binding
Nuclear division
Mitotic cell cycle
G1/S phase of cell cycle
RG4 [10-E]
Hmgb2
Cenpe
Birc5
Cdc20
Tpx2
Microtubule binding
Mitotic anaphase
Microglia [20-E]
Fos
Jun
Ly6e
Mpeg1
Ccl3
Ccl4
Microglial activation
Smarca4
Yeats4
Eif4g1
Eif3d
Taf9
Eif4e2
Translation
Choroid plexus [22-E]
Enpp2
Kcne2
Ttr
Fabp7
Zbtb20
Choroid
Ependymal precursors

Supplementary Figure 18. Heatmap representation of cellular sub-clusters within E14.5 cell types. We identified cellular sub-clusters in 7 of the 22 E14.5 cell types, 3 of which are shown in Fig. 2. The expression of sub-cluster-specific genes was median-centered and hierarchically clustered for all cells within the greater cluster and expression values were then plotted as a heatmap. Some key genes of interest and functional ontologies/pathways into which these genes fall are shown. Marker genes that describe the entire cluster or other genes expression in >75% or <25% of cells are not shown.

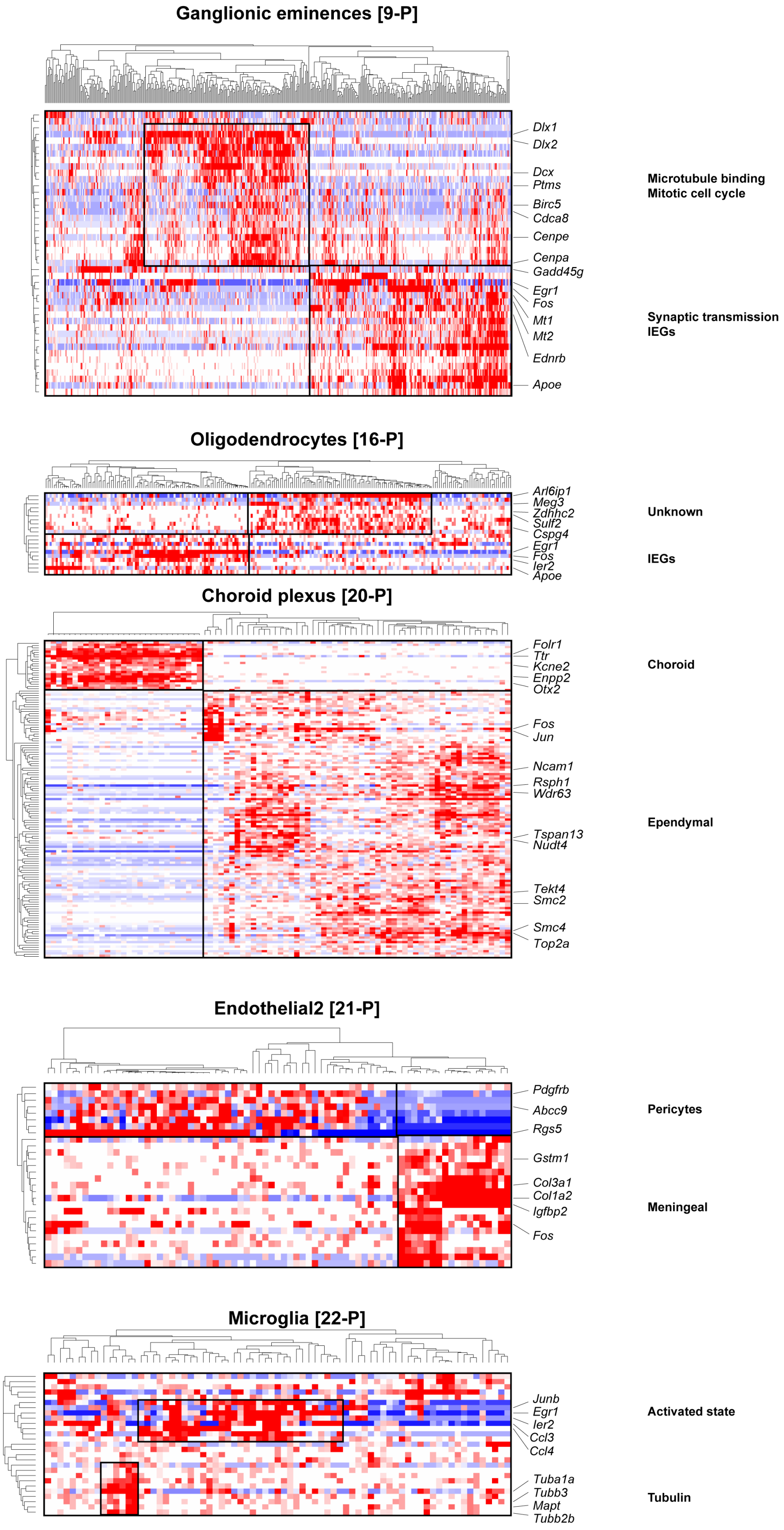
Ganglionic eminences [9-P]
Dlx1
Dlx2
Dcx
Ptms
Birc5
Cdca8
Cenpe
Cenpa
Gadd45g
Egr1
Fos
Mt1
Mt2
Ednrb
Apoe
Microtubule binding
Mitotic cell cycle
Synaptic transmission
IEGs
Oligodendrocytes [16-P]
Arl6ip1
Meg3
Zdhhc2
Sulf2
Cspg4
Egr1
Fos
Ier2
Apoe
Unknown
IEGs
Choroid plexus [20-P]
Folr1
Ttr
Kcne2
Enpp2
Otx2
Fos
Jun
Ncam1
Rsph1
Wdr63
Tspan13
Nudt4
Tekt4
Smc2
Smc4
Top2a
Choroid
Ependymal
Endothelial2 [21-P]
Pdgfrb
Abcc9
Rgs5
Gstm1
Col3a1
Col1a2
Igfbp2
Fos
Pericytes
Meningeal
Microglia [22-P]
Junb
Egr1
Ier2
Ccl3
Ccl4
Tuba1a
Tubb3
Mapt
Tubb2b
Activated state
Tubulin

Supplementary Figure 19. Heatmap representation of cellular sub-clusters within P0 cell types. We identified cellular sub-clusters in 5 of the 22 P0 cell types. The expression of sub-cluster-specific genes was median-centered and hierarchically clustered for all cells within the greater cluster and expression values were then plotted as a heatmap. Some key genes of interest and functional ontologies/pathways into which these genes fall are shown. Marker genes that describe the entire cluster or other genes expression in >75% or <25% of cells are not shown.

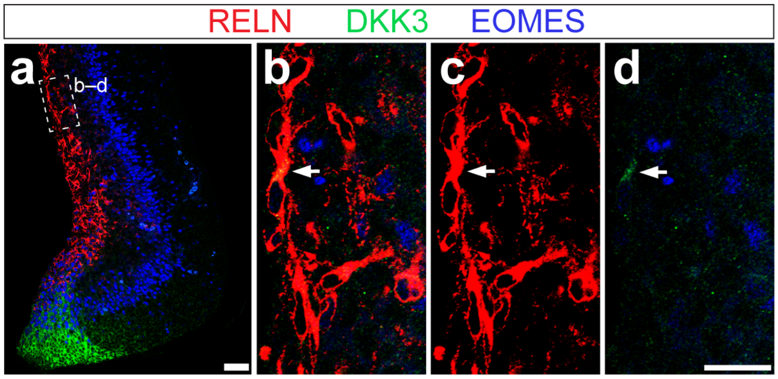

Supplementary Figure 20. Empirical validation of Layer I sub-clusters in the E14.5 cortex. Co-staining with DKK3 (green), EOMES (TBR2, blue), and RELN (Reelin, red). Cells expressing both DKK3 and RELN are labeled with white arrows. Scale bar represents 20 μm.

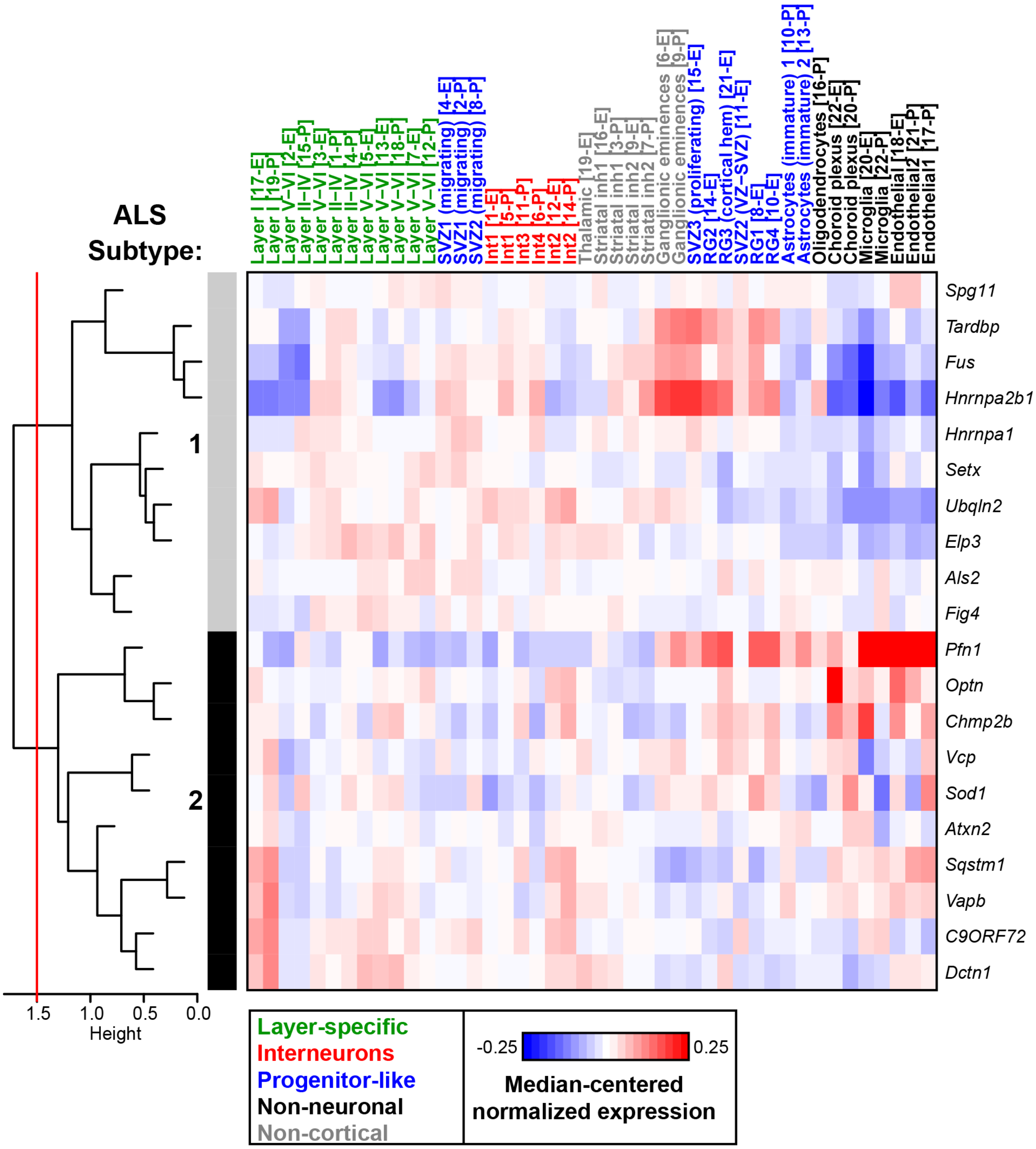

Supplementary Figure 21. Genes linked to amyotrophic lateral sclerosis (ALS) form two subtypes based on cortical expression patterns. Heatmap of ALS-linked genes clustered hierarchically by their expression across all 22 cell types for both E14.5 and P0. Pearson correlation distances were used to draw the dendrogram, and a height threshold of 1.5 (red line) was used to identify potential subtypes.

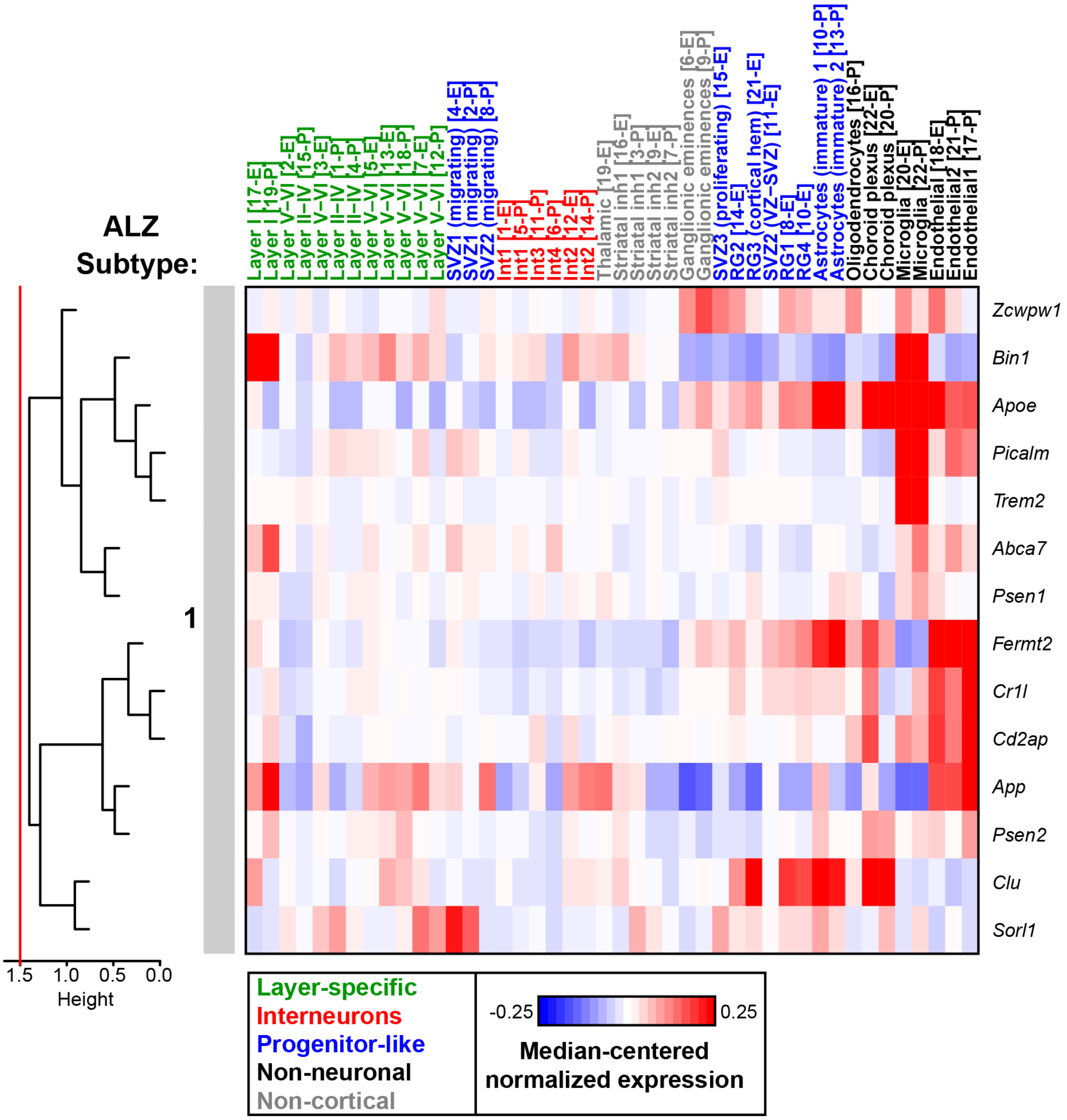

Supplementary Figure 22. Genes linked to Alzheimer's disease (ALZ) form one subtype based on cortical expression patterns. Heatmap of Alzheimer's-linked genes clustered hierarchically by their expression across all 22 cell types for both E14.5 and P0. Pearson correlation distances were used to draw the dendrogram, and a height threshold of 1.5 (red line) was used to identify potential subtypes.

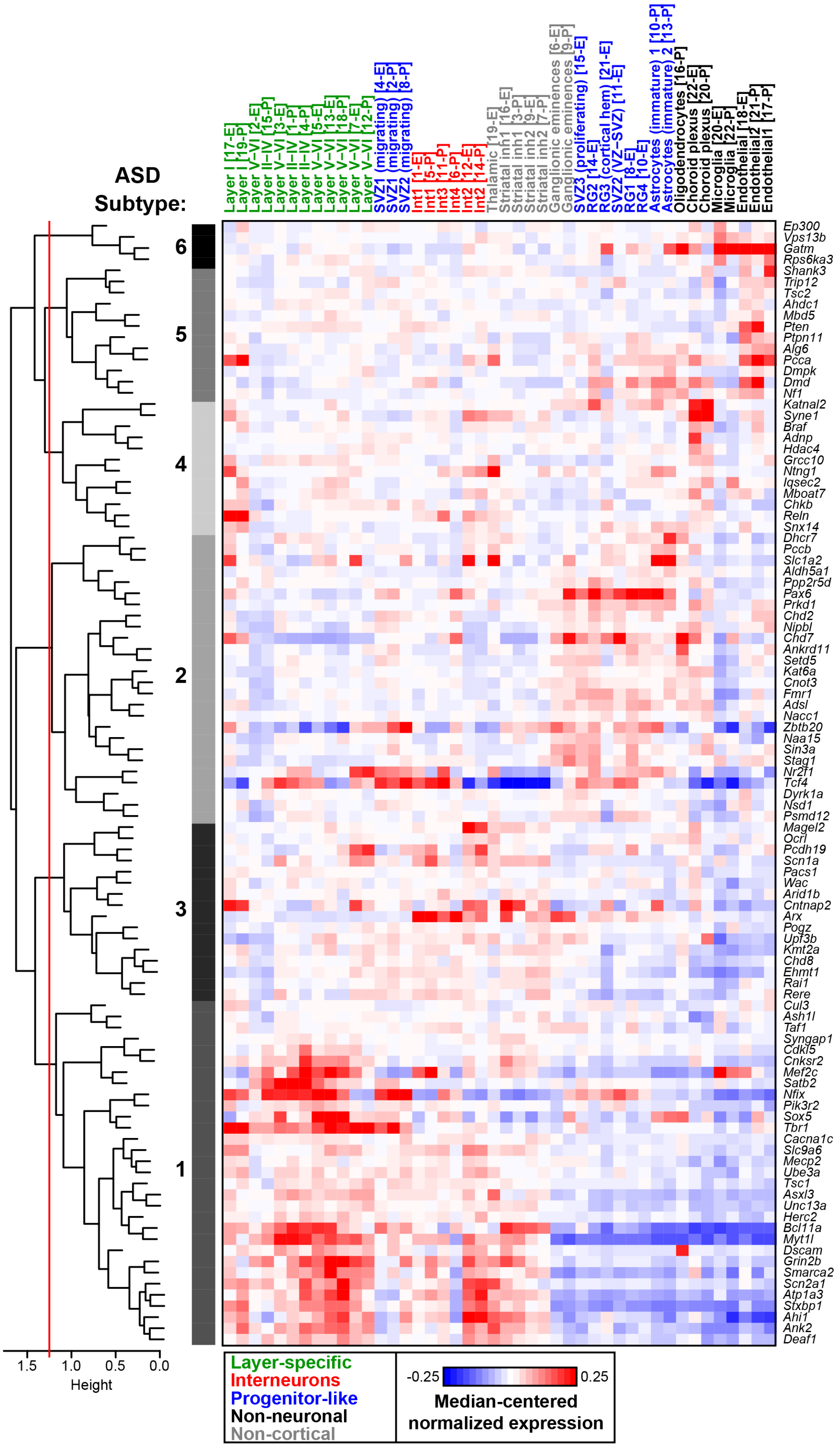
ASD Subtype:
6
5
4
2
3
1
1.5
1.0
0.5
0.0
Height
Layer I [17-E]
Layer I [19-P]
Layer V–VI [2-E]
Layer II–IV [15-P]
Layer V–VI [3-E]
Layer II–IV [1-P]
Layer II–IV [4-P]
Layer V–VI [5-E]
Layer V–VI [13-E]
Layer V–VI [18-P]
Layer V–VI [7-E]
Layer V–VI [12-P]
SVZ1 (migrating) [4-E]
SVZ1 (migrating) [2-P]
SVZ2 (migrating) [8-P]
Int1 [1-E]
Int1 [5-P]
Int3 [11-P]
Int4 [6-P]
Int2 [12-E]
Int2 [14-P]
Thalamic [19-E]
Striatal inh1 [16-E]
Striatal inh1 [3-P]
Striatal inh2 [9-E]
Striatal inh2 [7-P]
Ganglionic eminences [6-E]
Ganglionic eminences [9-P]
SVZ3 (proliferating) [15-E]
RG2 [14-E]
RG3 (cortical hem) [21-E]
SVZ2 (VZ–SVZ) [11-E]
RG1 [8-E]
RG4 [10-E]
Astrocytes (immature) 1 [10-P]
Astrocytes (immature) 2 [13-P]
Oligodendrocytes [16-P]
Choroid plexus [22-E]
Choroid plexus [20-P]
Microglia [20-E]
Microglia [22-P]
Endothelial [18-E]
Endothelial2 [21-P]
Endothelial1 [17-P]
Ep300
Vps13b
Gatm
Rps6ka3
Shank3
Trip12
Tsc2
Ahdc1
Mbd5
Pten
Ptpn11
Alg6
Pcca
Dmpk
Dmd
Nf1
Katnal2
Syne1
Braf
Adnp
Hdac4
Grcc10
Ntng1
Iqsec2
Mboat7
Chkb
Reln
Snx14
Dhcr7
Pccb
Slc1a2
Aldh5a1
Ppp2r5d
Pax6
Prkd1
Chd2
Nipbl
Chd7
Ankrd11
Setd5
Kat6a
Cnot3
Fmr1
Adsl
Nacc1
Zbtb20
Naa15
Sin3a
Stag1
Nr2f1
Tcf4
Dyrk1a
Nsd1
Psmd12
Magel2
Ocrl
Pcdh19
Scn1a
Pacs1
Wac
Arid1b
Cntnap2
Arx
Pogz
Upf3b
Kmt2a
Chd8
Ehmt1
Rai1
Rere
Cul3
Ash1l
Taf1
Syngap1
Cdkl5
Cnksr2
Mef2c
Satb2
Nfix
Pik3r2
Sox5
Tbr1
Cacna1c
Slc9a6
Mecp2
Ube3a
Tsc1
Asxl3
Unc13a
Herc2
Bcl11a
Myt1l
Dscam
Grin2b
Smarca2
Scn2a1
Atp1a3
Stxbp1
Ahi1
Ank2
Deaf1
Layer-specific
Interneurons
Progenitor-like
Non-neuronal
Non-cortical
-0.25
0.25
Median-centered normalized expression

Supplementary Figure 23. Genes linked to autism spectrum disorder (ASD) form six subtypes based on cortical expression patterns. Heatmap of ASD-linked genes clustered hierarchically by their expression across all 22 cell types for both E14.5 and P0. Pearson correlation distances were used to draw the dendrogram, and a height threshold of 1.25 (red line) was used to identify potential subtypes.

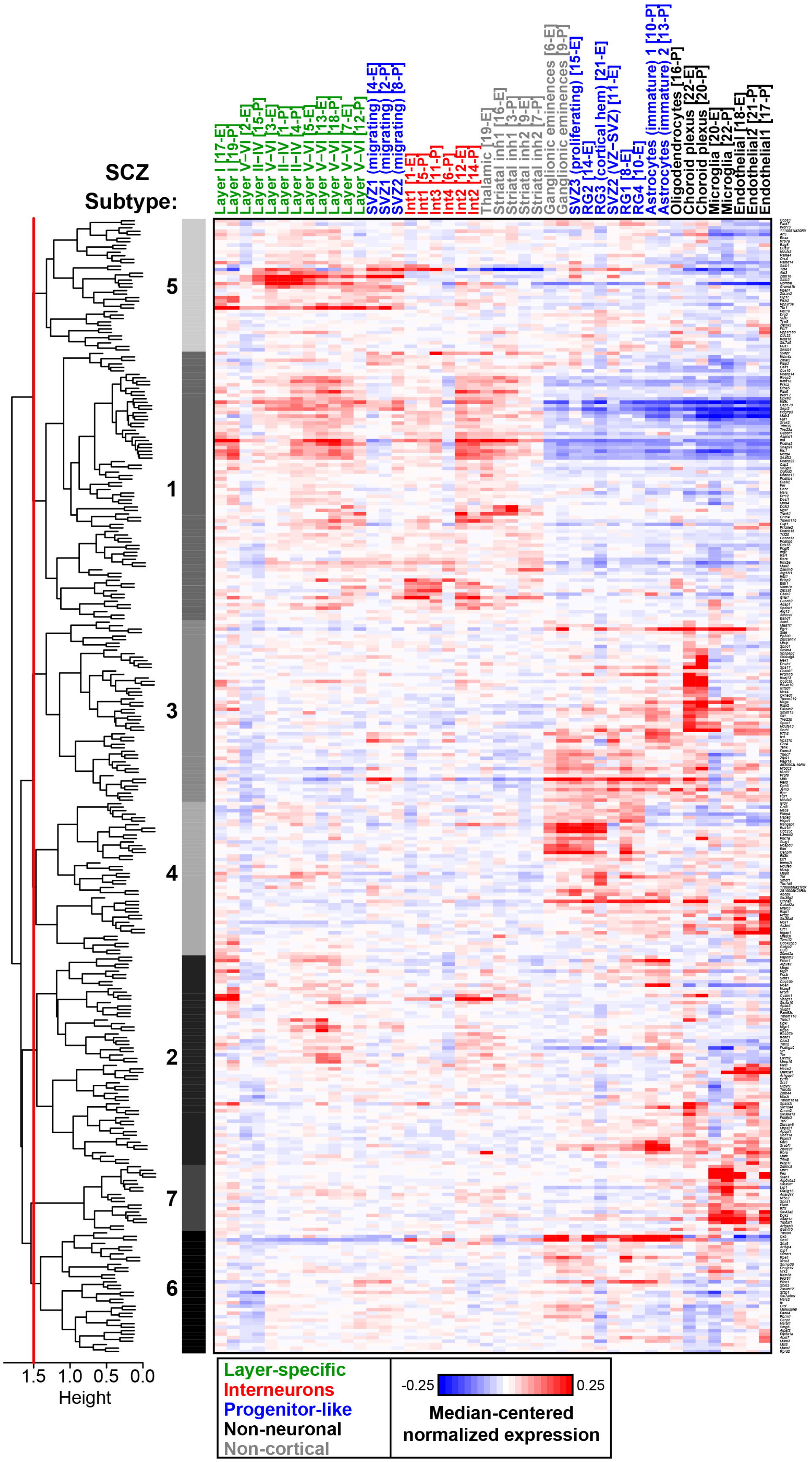
SCZ
Subtype:
5
1
3
4
2
7
6
1.5 1.0 0.5 0.0
Height
Layer I [17-E]
Layer I [19-P]
Layer V–VI [2-E]
Layer II–IV [15-P]
Layer V–VI [3-E]
Layer II–IV [1-P]
Layer II–IV [4-P]
Layer V–VI [5-E]
Layer V–VI [13-E]
Layer V–VI [18-P]
Layer V–VI [7-E]
Layer V–VI [12-P]
SVZ1 (migrating) [4-E]
SVZ1 (migrating) [2-P]
SVZ2 (migrating) [8-P]
Int1 [1-E]
Int1 [5-P]
Int3 [11-P]
Int4 [6-P]
Int2 [12-E]
Int2 [14-P]
Thalamic [19-E]
Striatal inh1 [16-E]
Striatal inh1 [3-P]
Striatal inh2 [9-E]
Striatal inh2 [7-P]
Ganglionic eminences [6-E]
Ganglionic eminences [9-P]
SVZ3 (proliferating) [15-E]
RG2 [14-E]
RG3 (cortical hem) [21-E]
SVZ2 (VZ–SVZ) [11-E]
RG1 [8-E]
RG4 [10-E]
Astrocytes (immature) 1 [10-P]
Astrocytes (immature) 2 [13-P]
Oligodendrocytes [16-P]
Choroid plexus [22-E]
Choroid plexus [20-P]
Microglia [20-E]
Microglia [22-P]
Endothelial [18-E]
Endothelial2 [21-P]
Endothelial1 [17-P]
Layer-specific
Interneurons
Progenitor-like
Non-neuronal
Non-cortical
-0.25
0.25
Median-centered normalized expression

Supplementary Figure 24. Genes linked to schizophrenia (SCZ) form seven subtypes based on cortical expression patterns. Heatmap of SCZ-linked genes clustered hierarchically by their expression across all 22 cell types for both E14.5 and P0. Pearson correlation distances were used to draw the dendrogram, and a height threshold of 1.5 (red line) was used to identify potential subtypes.

Sample	Age	Raw Number Mouse Cells	Raw Number Human Cells	Raw Number Mixed Cells	Final Number Mouse Cells	Number Mouse Reads
E14.WT1	E14.5	2409	26	58	2376	98351631
E14.WT2	E14.5	2959	27	13	2938	131371970
E14.WT3	E14.5	1426	23	46	1389	50853367
E14.WT4	E14.5	897	13	86	866	55918810
E14.WT5	E14.5	1441	27	32	1434	52825003
E14.WT6	E14.5	1937	32	31	1928	89056846
P0.WT1	P0	2443	35	21	2419	144389203
P0.WT2	P0	2896	66	38	2888	128999337
P0.WT3	P0	2349	53	97	2307	116787018

Supplementary Table 1. Sample metadata. Total numbers of reads and cells for each biological replicate
