# Supplementary material for: Single-cell transcriptomic analysis of mouse neocortical development
Source: Nat Commun. 2019 Jan 11;10:134. doi: 10.1038/s41467-018-08079-9 (PMC6329831; doi:10.1038/s41467-018-08079-9)
Supplement: Supplementary file 2 — Description of Additional Supplementary Files [file 41467_2018_8079_MOESM2_ESM.pdf]

Description of Additional Supplementary Files

File Name: Supplementary Data 1
Description: Collapsed gene expression values for all cell types. Normalized expression values were collapsed within cell type (using mean). Genes detected in only one age contain NA value for cell types with undetected expression.

File Name: Supplementary Data 2
Description: Comprehensive cell type annotation and validation. All cell type annotations, along with additional references confirming the identity of these cell types and their marker genes, as well as pathway-level enrichment analyses that describe the predominant transcriptional signatures of each cell type.
