# Supplementary material for: Single-cell transcriptomic analysis of mouse neocortical development
Source: Nat Commun. 2019 Jan 11;10:134. doi: 10.1038/s41467-018-08079-9 (PMC6329831; doi:10.1038/s41467-018-08079-9)
Supplement: Supplementary file 6 — Reporting Summary [file 41467_2018_8079_MOESM6_ESM.pdf]

nature research

Corresponding author(s): Mark Zylka

Reporting Summary

Nature Research wishes to improve the reproducibility of the work that we publish. This form provides structure for consistency and transparency in reporting. For further information on Nature Research policies, see Authors & Referees and the Editorial Policy Checklist.

Statistical parameters

When statistical analyses are reported, confirm that the following items are present in the relevant location (e.g. figure legend, table legend, main text, or Methods section).

n/a	Confirmed	
☐	☒	The exact sample size (n) for each experimental group/condition, given as a discrete number and unit of measurement
☐	☒	An indication of whether measurements were taken from distinct samples or whether the same sample was measured repeatedly
☐	☒	The statistical test(s) used AND whether they are one- or two-sided<br>*Only common tests should be described solely by name; describe more complex techniques in the Methods section.*
☒	☐	A description of all covariates tested
☐	☒	A description of any assumptions or corrections, such as tests of normality and adjustment for multiple comparisons
☒	☐	A full description of the statistics including central tendency (e.g. means) or other basic estimates (e.g. regression coefficient) AND variation (e.g. standard deviation) or associated estimates of uncertainty (e.g. confidence intervals)
☒	☐	For null hypothesis testing, the test statistic (e.g. F, t, r) with confidence intervals, effect sizes, degrees of freedom and P value noted<br>*Give P values as exact values whenever suitable.*
☒	☐	For Bayesian analysis, information on the choice of priors and Markov chain Monte Carlo settings
☒	☐	For hierarchical and complex designs, identification of the appropriate level for tests and full reporting of outcomes
☒	☐	Estimates of effect sizes (e.g. Cohen's d, Pearson's r), indicating how they were calculated
☒	☐	Clearly defined error bars<br>*State explicitly what error bars represent (e.g. SD, SE, CI)*

Our web collection on statistics for biologists may be useful.

Software and code

Policy information about availability of computer code

Data collection	bcl2fastq version 2.18.0.12
Data analysis	STAR 2.5.2b<br>Picard Tools 2.2.4<br>DropSeq Toolkit 1.12

For manuscripts utilizing custom algorithms or software that are central to the research but not yet described in published literature, software must be made available to editors/reviewers upon request. We strongly encourage code deposition in a community repository (e.g. GitHub). See the Nature Research guidelines for submitting code & software for further information.

Data

Policy information about availability of data

All manuscripts must include a data availability statement. This statement should provide the following information, where applicable:

- Accession codes, unique identifiers, or web links for publicly available datasets
- A list of figures that have associated raw data
- A description of any restrictions on data availability

The web-based visualization tool described above is available at http://zylkalab.org/data. All code for data analysis, including cluster identification and refinement,

cellular sub-clustering analysis, and unprocessed gene expression matrices are available on GitHub at https://github.com/jeremymsimon/MouseCortex. Raw and processed data were also deposited to the Gene Expression Omnibus under accession 'GSE123335[https://www.ncbi.nlm.nih.gov/geo/query/acc.cgi?acc=GSE123335]'.

Field-specific reporting

Please select the best fit for your research. If you are not sure, read the appropriate sections before making your selection.

☒ Life sciences ☐ Behavioural & social sciences ☐ Ecological, evolutionary & environmental sciences

For a reference copy of the document with all sections, see nature.com/authors/policies/ReportingSummary-flat.pdf

Life sciences study design

All studies must disclose on these points even when the disclosure is negative.

Sample size	No pre-determination of sample size was performed or applicable. We targeted roughly 10,000 cells per age. We sufficiently captured the known cellular diversity of the cortex at each age, and identified known/novel subtypes, states, and trajectories.
Data exclusions	There were no data exclusions
Replication	Cell types and states were validated using online imaging repositories and/or immunofluorescence as shown in the manuscript
Randomization	N/A to this study, though all samples were sequenced on one high-throughput sequencing flowcell to reduce batch effects
Blinding	N/A to this study as there are no relevant sample groups

Reporting for specific materials, systems and methods

Materials & experimental systems

n/a	Involved in the study	
☒	☐	Unique biological materials
☐	☒	Antibodies
☒	☐	Eukaryotic cell lines
☒	☐	Palaeontology
☐	☒	Animals and other organisms
☒	☐	Human research participants

Methods

n/a	Involved in the study	
☒	☐	ChIP-seq
☒	☐	Flow cytometry
☒	☐	MRI-based neuroimaging

Antibodies

Antibodies used	Primary antibodies used were mouse anti-Reelin (Millipore, MAB5364; IgG1) at 1:1000 dilution, rabbit anti-DKK3 (Abcam, ab2459) at 1:500 dilution, mouse anti-SOX2 (R&D System, MAB2018; IgG2a) at 1:500 dilution, rat anti-TBR2 (eBioscience, 14-4879-12) at 1:500 dilution), rabbit anti-CRE (#13056S, Cell Signaling) at 1:500 dilution and goat anti-CALB2 (Swant, CG1) at 1:500 dilution. Secondary antibodies used were goat anti-mouse IgG1 (Alexa 488 or 647; Thermo Scientific), goat anti-mouse IgG2a (Alexa 488, Thermo Scientific), donkey anti-rabbit IgG (Alexa 488, 568 or 647, Thermo Scientific), donkey anti-goat IgG (Alexa 568, Thermo Scientific) and donkey anti-Rat IgG (Cy3, Jackson ImmunoResearch). All secondary antibodies were diluted 1:1000 in blocking buffer.
Validation	MAB5364: validated for IHC and WB, as per manufacturer<br>ab2459: validated for ICC/IF, as per manufacturer<br>MAB2018: validated for ICC/WB, as per manufacturer<br>CG1: validated for IHC, as per manufacturer

Animals and other organisms

Policy information about studies involving animals; ARRIVE guidelines recommended for reporting animal research

Laboratory animals	Mus musculus, C57BL/6J, E14.5 and P0, mixed male and female as noted

Wild animals	N/A
Field-collected samples	N/A
